# Supplementary material for: Script Concordance Tests for Formative Clinical Reasoning and Problem-Solving Assessment in General Pediatrics
Source: MedEdPORTAL. 2022 Sep 20;18:11274. doi: 10.15766/mep_2374-8265.11274 (PMC9485313; doi:10.15766/mep_2374-8265.11274)
Supplement: Supplementary file 1 — SCTs Without Answers.docxSCTs With Expert Answers.pdfScoring Guide.docxScoring Spreadsheet.xslx [file mep_2374-8265.11274-s001.zip › C. Scoring Guide.docx]

**SCT Scoring Guide**

Instructions for scoring each SCT using Bland et al’s absolute distance method is detailed below. Appendix D will automatically use expert answers from Appendix B and calculate scores for each SCT for you, without the need for extra calculations. Should you wish to read more about different styles of scoring SCTs, please refer to Bland et al’s paper, attached below.:

1. Export your student’s data from your learning system into an excel file and assign student responses to de-identified aliases should you wish to eventually match SCT scores to de-identified summative assessment scores. Alternatively, if you administered the SCT off-line, manually input each student’s answer choices into an excel file.
2. Convert student scores from a 5-point Likert scale to a 3-point Likert scale. As detailed above, this means changing answers from +2 to +1 and -2 to -1.
3. Input student answer choices into the designated columns in Appendix D. Our spreadsheet will automatically calculate student’s percent score using our expert responses found in Appendix B. Percentage score will automatically populate as well.

*Note: Disregard current scores in each column. These scores correlate to an answer of “0” and will change when you input your own data.*


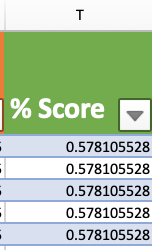

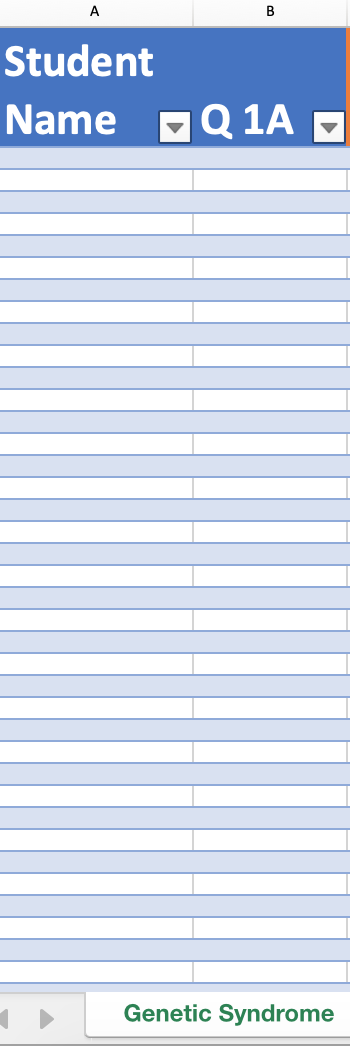


Score will auto populate here.

Insert -1,0, or 1 score here.

1. Inputting student answer choices will also allow you to view descriptive statistics for each individual SCT in Appendix D.


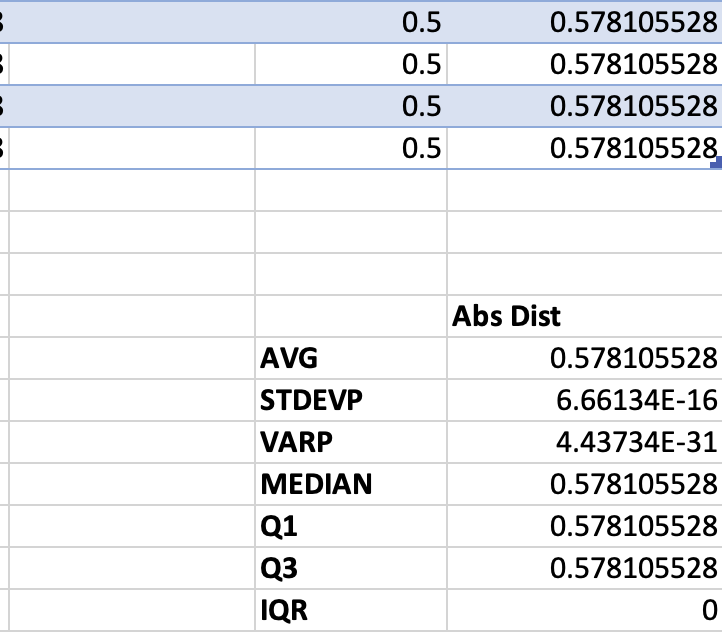


1. You may now use these SCT scores to outcomes to other summative assessments, as we did in our study. You may do so by using another spreadsheet to associate scores with SCT outcomes. This second excel spreadsheet can be uploaded to your data analysis software of your choice (ie: SPSS, RStudio, etc.) for further analysis.

Helpful Links:

Bland AC, Kreiter CD, Gordon JA. The Psychometric Properties of Five Scoring Methods Applied to the Script Concordance Test. *Acad Med*. 2005;80(4):395-399.

<https://www.tandfonline.com/doi/full/10.1080/10401334.2014.884464>
