# Supplementary material for: Script Concordance Tests for Formative Clinical Reasoning and Problem-Solving Assessment in General Pediatrics
Source: MedEdPORTAL. 2022 Sep 20;18:11274. doi: 10.15766/mep_2374-8265.11274 (PMC9485313; doi:10.15766/mep_2374-8265.11274)
Supplement: Supplementary file 1 — SCTs Without Answers.docxSCTs With Expert Answers.pdfScoring Guide.docxScoring Spreadsheet.xslx [file mep_2374-8265.11274-s001.zip › A. SCTs Without Answers.docx]

**Genetic Syndrome**

**You are called to the newborn nursery by the labor and delivery nurse to examine a newborn infant with abnormal tone, dysmorphic facial features and a heart murmur.**

| If you were considering a diagnosis of: | And then you find: | Less likely |  | This diagnosis  becomes: |  | More likely |
| --- | --- | --- | --- | --- | --- | --- |
| Q1A. Trisomy 21 | Epicanthal folds | -2 | -1 | 0 | +1 | +2 |
| Q1B. Prenatal Alcohol Exposure | A smooth philtrum | -2 | -1 | 0 | +1 | +2 |
| Q1C. Trisomy 18 | Macrocephaly | -2 | -1 | 0 | +1 | +2 |

**You elicit further prenatal history from the mother’s chart, perform a complete physical examination on the infant and consider your further evaluation for this infant.**

| If you were considering ordering a: | And then on examination you find: | Less likely |  | This investigation  becomes: |  | More likely |
| --- | --- | --- | --- | --- | --- | --- |
| Q2A. Renal Ultrasound | A single umbilical artery | -2 | -1 | 0 | +1 | +2 |
| Q2B. A karyotype (cytogenetic study) | Brushfield's spots | -2 | -1 | 0 | +1 | +2 |
| Q2C. An echocardiogram | A normal cardiac exam | -2 | -1 | 0 | +1 | +2 |

**A karyotype is obtained and confirms a diagnosis of Trisomy 21. The parents inquire about possible future complications from this diagnosis. In children with Trisomy 21, how would you handle the following associated issues in the future?**

| If you were thinking of: | And then you find: | Less likely |  | This management  becomes: |  | More likely |
| --- | --- | --- | --- | --- | --- | --- |
| Q3A. Starting the patient on levothyroxine | A high serum TSH level on routine health supervision labs | -2 | -1 | 0 | +1 | +2 |
| Q3B. Referring the patient to a neurosurgeon | A normal cervical spine screening series for atlantoaxial instability | -2 | -1 | 0 | +1 | +2 |
| Q3C. Ordering a complete blood count (CBC) with differential and blood smear | A scattered petechial rash | -2 | -1 | 0 | +1 | +2 |

**Rash**

**A five-year child presents to his primary care provider with a rash on his upper and lower extremities for 2 weeks.**

| **If you were thinking of a diagnosis of:** | **And then you find:** | **Less likely** |  | **This diagnosis**  **becomes:** |  | **More likely** |
| --- | --- | --- | --- | --- | --- | --- |
| Q1A. Atopic Dermatitis | A past medical history of allergic rhinitis and asthma | -2 | -1 | 0 | +1 | +2 |
| Q1B. Impetigo | Honey colored crusting on erythematous plaques in the popliteal creases | -2 | -1 | 0 | +1 | +2 |
| Q1C. Erythema Multiforme | A maculopapular rash involving the trunk and face | -2 | -1 | 0 | +1 | +2 |

**You elicit further history from the parents. The patient has had intermittent dry rough skin since birth. In infancy, the rash primarily presented on his cheeks and trunk, but for the last few years, he has had intermittent outbreaks of rough skin on his trunk and popliteal and antecubital fossae.**

| **If you were considering the following laboratory test:** | **And then you find:** | **Less likely** |  | **This investigation**  **becomes:** |  | **More likely** |
| --- | --- | --- | --- | --- | --- | --- |
| Q2A. KOH preparation for microscopic examination | Scattered circular or ovoid lesions with no flaking or central clearing | -2 | -1 | 0 | +1 | +2 |
| Q2B. A rapid streptococcal antigen test | A rough popular truncal rash and axillary temperature of 99.6F | -2 | -1 | 0 | +1 | +2 |
| Q2C. A wound culture | A tender area of erythematous skin with overlying honey colored crusting | -2 | -1 | 0 | +1 | +2 |

**You conduct a KOH preparation on one of the circular lesions which is negative for fungal elements. However, you notice the patient keeps itching the affected areas. His mother remembers he recently starting bathing with a scented bath soap and inquires if this could possibly be triggering the recent flare in his symptoms.**

| **If you were thinking of:** | **And then you find:** | **Less likely** |  | **This treatment**  **becomes:** |  | **More likely** |
| --- | --- | --- | --- | --- | --- | --- |
| Q3A. Perscribing a low potency steroid cream | Multiple areas of skin breakdown in affected areas | -2 | -1 | 0 | +1 | +2 |
| Q3B. Refering the patient to pediatric dermatology | The child has failed previous courses of topical steroids and immune suppressants | -2 | -1 | 0 | +1 | +2 |
| Q3C. Initiating antibiotic therapy | The patient had a history of a MRSA abscess 1 month prior in an affected area. | -2 | -1 | 0 | +1 | +2 |

**Abdominal Mass**

**You are called by the newborn nursery nurse to examine 36 hours old full-term infant with a distended abdomen. You complete a physical examination on the infant.**

| **If you were thinking of a diagnosis of:** | **And then you find:** | **Less likely** |  | **This diagnosis**  **becomes:** |  | **More likely** |
| --- | --- | --- | --- | --- | --- | --- |
| Q1A. Dysplastic kidney | A right sided compressible flank mass | -2 | -1 | 0 | +1 | +2 |
| Q1B. An intestinal obstruction | Upward slanting palpebral fissures and a single palmar crease. | -2 | -1 | 0 | +1 | +2 |
| Q1C. Distended bladder | A sacral dimple with an overlying hair tuft | -2 | -1 | 0 | +1 | +2 |

**You elicit further history from the infant’s mother and father and they report no history of abdominal conditions or pathologies. You also discover the infant’s prenatal course was unremarkable.**

| **If you were considering the following diagnostic study:** | **And then you find:** | **Less likely** |  | **This investigation**  **becomes:** |  | **More likely** |
| --- | --- | --- | --- | --- | --- | --- |
| Q2A. Ordering a blood culture | A history of poor feeding | -2 | -1 | 0 | +1 | +2 |
| Q2B. Ordering an abdominal radiograph | A history of one episode of bilious vomiting | -2 | -1 | 0 | +1 | +2 |
| Q2C. Ordering an abdominal ultrasound | Normal urine output | -2 | -1 | 0 | +1 | +2 |

**You discuss further management of the infant with your attending physician.**

| **If you were thinking of the following management:** | **And then you find:** | **Less likely** |  | **This treatment**  **becomes:** |  | **More likely** |
| --- | --- | --- | --- | --- | --- | --- |
| Q3A. Referring the patient to pediatric neurosurgery | Bladder distention on ultrasonography | -2 | -1 | 0 | +1 | +2 |
| Q3B. Initiating antibiotic therapy | Multiple renal cysts on ultrasonography | -2 | -1 | 0 | +1 | +2 |
| Q3C. Inserting a nasogastric tube | A double bubble sign on the abdominal radiograph | -2 | -1 | 0 | +1 | +2 |

**Diarrhea**

**A 12-month-old male child presents to the pediatric emergency department with a three-day history of diarrhea and intermittent abdominal pain.**

| If you were thinking of a diagnosis of: | And then you find: | **Less likely** |  | **This diagnosis**  **becomes:** |  | **More likely** |
| --- | --- | --- | --- | --- | --- | --- |
| Q1A. Viral gastroenteritis | A history of vomiting multiple times in the past 24 hours | -2 | -1 | 0 | +1 | +2 |
| Q1B. Bacterial gastroenteritis | A few specks of blood in his stool | -2 | -1 | 0 | +1 | +2 |
| Q1C. Lactose intolerance | A temperature of 38.1°C rectally | -2 | -1 | 0 | +1 | +2 |

**During your physical examination, you elicit further history from the mother. She reports that the child attends day care and numerous peers have been sent home with similar symptoms.**

| If you were considering ordering the following study: | And then you find a: | **Less likely** |  | **This investigation**  **becomes:** |  | **More likely** |
| --- | --- | --- | --- | --- | --- | --- |
| Q2A. Serum electrolytes | Urine dip positive for ketones | -2 | -1 | 0 | +1 | +2 |
| Q2B. Abdominal radiograph | A burp cloth with a green tinge to his emesis | -2 | -1 | 0 | +1 | +2 |
| Q2C. Bacterial cultures of stool | A history of a pet snake in the home | -2 | -1 | 0 | +1 | +2 |

**You discover dry mucous membranes and tachycardia on exam. Due to concerns for dehydration, you obtain an electrolyte panel which is normal. The abdominal radiograph showed no evidence of obstruction.**

| If you were thinking of: | And then you find: | **Less likely** |  | **This treatment**  **becomes:** |  | **More likely** |
| --- | --- | --- | --- | --- | --- | --- |
| Q3A. Sending the child home with directions for oral rehydration therapy | A blood hematocrit of 40% and Hgb 12.0/dl on the complete blood count. | -2 | -1 | 0 | +1 | +2 |
| Q3B. Initiating intravenous hydration | The child has lost 10% of his baseline weight in the last two days | -2 | -1 | 0 | +1 | +2 |
| Q3C. Initiating antibiotic therapy | His stool for PMN’s (lactoferrin) is negative | -2 | -1 | 0 | +1 | +2 |

**Lymphadenopathy**

**A mother brings in her three-year old daughter to your office due to a one-week history of neck swelling.**

| **If you were thinking of a diagnosis of:** | **And then you find:** | **Less likely** |  | **This diagnosis**  **becomes:** |  | **More likely** |
| --- | --- | --- | --- | --- | --- | --- |
| Q1A. Strep pharyngitis | An erythematous pharynx with enlarged anterior cervical lymph nodes. | -2 | -1 | 0 | +1 | +2 |
| Q1B. Lymphadenitis | Multiple firm painless posterior cervical lymph nodes along the child’s left neck area | -2 | -1 | 0 | +1 | +2 |
| Q1C. Brachial cleft cyst | A single 3cm flocculent mass on the anterior aspect right side of the neck | -2 | -1 | 0 | +1 | +2 |

**You elicit further history from the mother. She notes the patient has had intermittent fevers for the past week.**

| **If you were considering the following study:** | **And then you find:** | **Less likely** |  | **This investigation**  **becomes:** |  | **More likely** |
| --- | --- | --- | --- | --- | --- | --- |
| Q2A. Epstein Barr titers (EBV IgG, IgM) | The child’s has been increasingly tired over the past three weeks | -2 | -1 | 0 | +1 | +2 |
| Q2B. Serum C-reactive protein levels | The fevers are typically 39.5ºC and the mother describes persistent redness of the posterior pharynx | -2 | -1 | 0 | +1 | +2 |
| Q2C. Biopsy of an enlarged lymph node | A history of two cats in the house. | -2 | -1 | 0 | +1 | +2 |

**You complete a full physical examination of the child and consider further management options.**

| **If you were thinking of the following management:** | **And then you find:** | **Less likely** |  | **This recommendation**  **becomes:** |  | **More likely** |
| --- | --- | --- | --- | --- | --- | --- |
| Q3A. Initiating intravenous immunoglobulin (IVIG) therapy | A diffuse erythematous truncal rash and swollen hands and feet. | -2 | -1 | 0 | +1 | +2 |
| Q3B. Performing a bone marrow biopsy. | A white blood count of 27,000/mm with 80% lymphocytes and a few (<5%) atypical cells. | -2 | -1 | 0 | +1 | +2 |
| Q3C. Starting antibiotic therapy | A 0.7cm erythematous papule on the forearm with enlarged tender axillary lymph nodes ipsilaterally. | -2 | -1 | 0 | +1 | +2 |

**Otalgia**

**A 2-year-old female has a 2-day history of left ear pain. Her mother has been giving her acetaminophen with temporary improvement in pain.**

| If you were thinking of a diagnosis of: | And then you find: | **Less likely** |  | **This diagnosis**  **becomes:** |  | **More likely** |
| --- | --- | --- | --- | --- | --- | --- |
| Q1A. Acute Otitis Media | A history of a runny nose and congestion | -2 | -1 | 0 | +1 | +2 |
| Q1B. Acute Otitis Externa | No history of recent swimming | -2 | -1 | 0 | +1 | +2 |
| Q1C. Foreign body in the external ear canal | A temperature of 38.5°C | -2 | -1 | 0 | +1 | +2 |

**You elicit further history from the mother. The mother reports that the patient has a history of multiple previous episodes of acute otitis media that have required oral antibiotics. You complete a physical exam on the patient.**

| If you were considering the following intervention: | And then you find: | **Less likely** |  | **This intervention**  **becomes:** |  | **More likely** |
| --- | --- | --- | --- | --- | --- | --- |
| Q2A. Ear canal irrigation | Bilateral myringotomy tubes on exam | -2 | -1 | 0 | +1 | +2 |
| Q2B. Complete blood count with differential | Purulent, erythematous tympanic membranes | -2 | -1 | 0 | +1 | +2 |
| Q2C. CT scan of head | Left-sided post-auricular swelling and erythema. | -2 | -1 | 0 | +1 | +2 |

**The patient’s mother inquiries about your management plans.**

| If you were thinking of: | And then you find: | **Less likely** |  | **This treatment**  **becomes:** |  | **More likely** |
| --- | --- | --- | --- | --- | --- | --- |
| Q3A. Prescribing oral amoxicillin for 10 days | An erythematous, swollen external ear canal with white discharge | -2 | -1 | 0 | +1 | +2 |
| Q3B. Supportive pain control | A mobile, non-tender, left tympanic membrane with a serous effusion | -2 | -1 | 0 | +1 | +2 |
| Q3C. ENT referral for bilateral myringotomy tube insertion | A history of two prior episodes of acute otitis media in the past 8 months | -2 | -1 | 0 | +1 | +2 |

**Vomiting**

**A sixteen-year-old female presents to her primary care provider with her parents for concerns of vomiting following meals for the past month.**

| **If you were thinking of a diagnosis of:** | **And then you discover:** | **Less likely** |  | **This diagnosis**  **becomes:** |  | **More likely** |
| --- | --- | --- | --- | --- | --- | --- |
| Q1A. Gastroesophageal reflux disease | A history of abdominal pain in the left lower quadrant with defecation | -2 | -1 | 0 | +1 | +2 |
| Q1B. Bulimia | The patient has an upcoming high school cheerleading tryout | -2 | -1 | 0 | +1 | +2 |
| Q1C. Pregnancy | The patient recently ended her last menstrual cycle 23 days ago | -2 | -1 | 0 | +1 | +2 |

**You elicit further history from the parents and the patient. The patient has recently been promoted to the varsity, competitive cheer squard. Due to her short stature and petite size, she has been selected to be a flyer and top to one of their stunt pyramids.**

| **If you were considering the following laboratory study:** | **And then you find:** | **Less likely** |  | **This investigation**  **becomes:** |  | **More likely** |
| --- | --- | --- | --- | --- | --- | --- |
| Q2A. A rapid urine HCG test | The patient denies sexual activity | -2 | -1 | 0 | +1 | +2 |
| Q2B. A electrocardiogram | A resting heartrate of 51 | -2 | -1 | 0 | +1 | +2 |
| Q2C. A prealbumin level | Lanugo on the patient’s trunk | -2 | -1 | 0 | +1 | +2 |

**After speaking to the adolescent privately, she admits to vomiting on purpose after meals and at times skipping meals to lose weight. She also admits she has been exercising 2-3 hours daily to assure she is in top form for her cheerleading team. She also admits to feeling overweight as compared to her cheerleading peers.**

| **If you were thinking of:** | **And then you discover:** | **Less likely** |  | **This management**  **becomes:** |  | **More likely** |
| --- | --- | --- | --- | --- | --- | --- |
| Q3A. Discussing your concerns of bulimia and anorexia with her parents | She has asked you to keep her behaviors in confidence | -2 | -1 | 0 | +1 | +2 |
| Q3B. Admitting her to the inpatient service to begin slow re-feeding | Her sodium level is 132 | -2 | -1 | 0 | +1 | +2 |
| Q3C. Referring her to an eating disorder expert in the psychiatry department | The patient does not feel she has a problem and promises to stop after the competitive cheer season | -2 | -1 | 0 | +1 | +2 |

**Fever Without a Source**

**A 3-week-old female infant presents to the pediatric emergency department with a 2-hour history of rectal temperature of 101.5F**

| **If you were thinking of:** | **And then you find:** | **Less likely** |  | **This diagnosis**  **becomes:** |  | **More likely** |
| --- | --- | --- | --- | --- | --- | --- |
| Q1A. Viral infection | bulging anterior fontanelle (or petchiae) | -2 | -1 | 0 | +1 | +2 |
| Q1B. Bacterial infection | copious clear nasal secretions | -2 | -1 | 0 | +1 | +2 |
| Q1C. HSV infection | vesicular rash on erythematous base | -2 | -1 | 0 | +1 | +2 |

**You elicit further history from the parents. The mother reports that the infant was born at 33 weeks gestation and was just released from the neonatal intensive care unit 3 days ago.**

| **If you were considering:** | **And then you find:** | **Less likely** |  | **This investigation**  **becomes:** |  | **More likely** |
| --- | --- | --- | --- | --- | --- | --- |
| Q2A. Ordering a urine culture  (Ordering a CBC with diff) | Urine dip negative for nitrates  (Maternal history of negative group B strep serology) | -2 | -1 | 0 | +1 | +2 |
| Q2B. Ordering a chest radiograph | Respirtaory rate of 62 at rest with no retractions | -2 | -1 | 0 | +1 | +2 |
| Q2C. Performing a lumbar puncture | The patient has a positive rapid test for respiratory syncytial virus | -2 | -1 | 0 | +1 | +2 |

**Blood, urine and CSF samples were sent to the laboratory for further evaluation and culture. The patient is admitted to the inpatient service.**

| **If you were thinking of:** | **And then you find:** | **Less likely** |  | **This treatment**  **becomes:** |  | **More likely** |
| --- | --- | --- | --- | --- | --- | --- |
| Q3A. Ordering a renal ultrasound | No growth of bacteria in the urine culture | -2 | -1 | 0 | +1 | +2 |
| Q3B. Starting an antiviral medication (acyclovir) | The child had a brief 1 minute seizure-like episode in the emergency department | -2 | -1 | 0 | +1 | +2 |
| Q3C. Initiating antibiotic therapy | Impaired renal function on basic metabolic panel  (Positive Viral PCR for influenza) | -2 | -1 | 0 | +1 | +2 |
