# Supplementary material for: Script Concordance Tests for Formative Clinical Reasoning and Problem-Solving Assessment in General Pediatrics
Source: MedEdPORTAL. 2022 Sep 20;18:11274. doi: 10.15766/mep_2374-8265.11274 (PMC9485313; doi:10.15766/mep_2374-8265.11274)
Supplement: Supplementary file 1 — SCTs Without Answers.docxSCTs With Expert Answers.pdfScoring Guide.docxScoring Spreadsheet.xslx [file mep_2374-8265.11274-s001.zip › B. SCTs With Expert Answers.pdf]

Genetic Syndrome

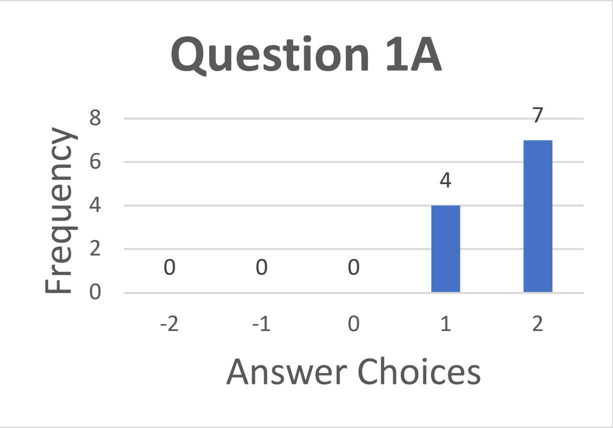

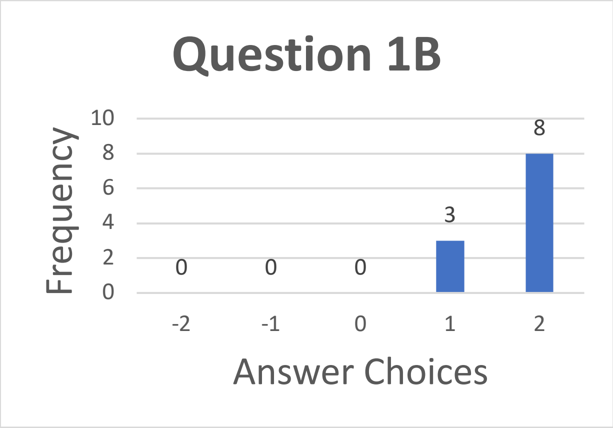

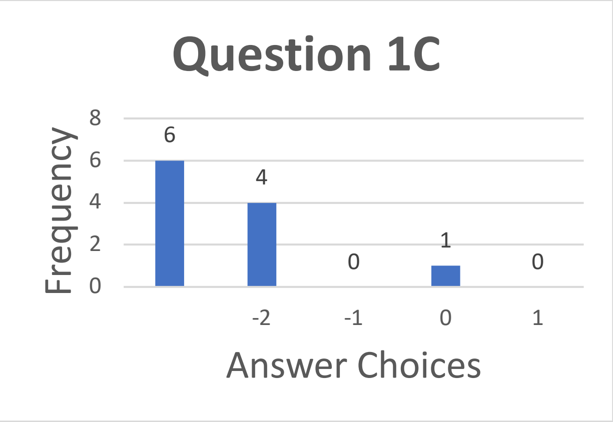

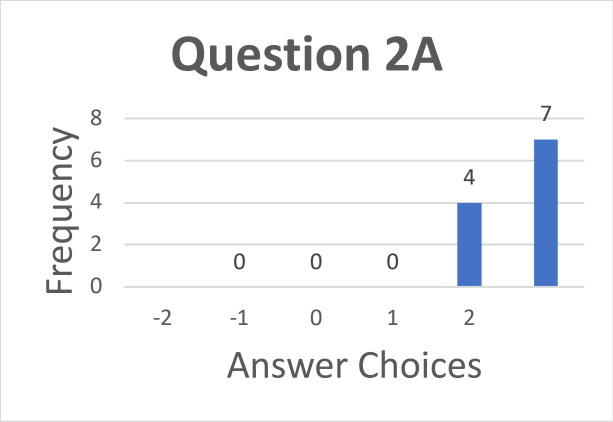

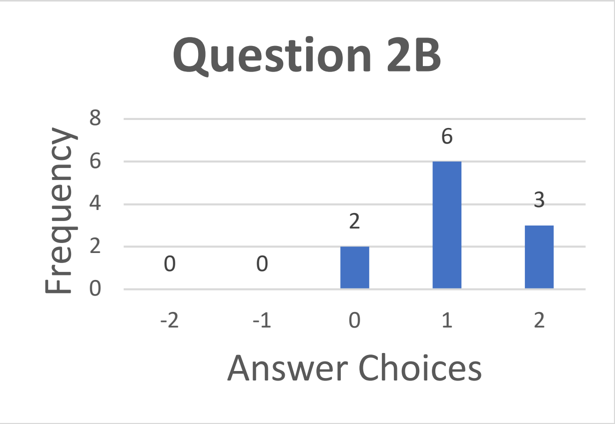

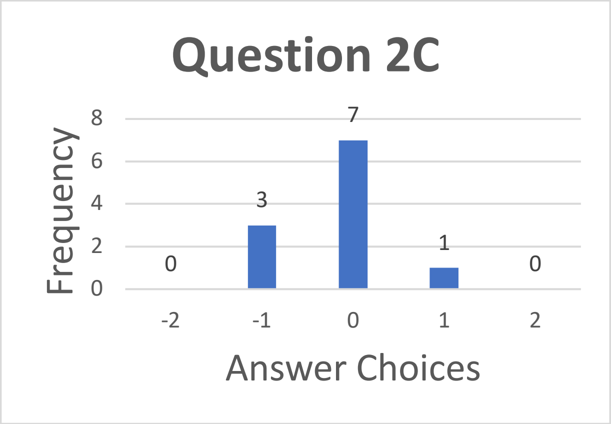

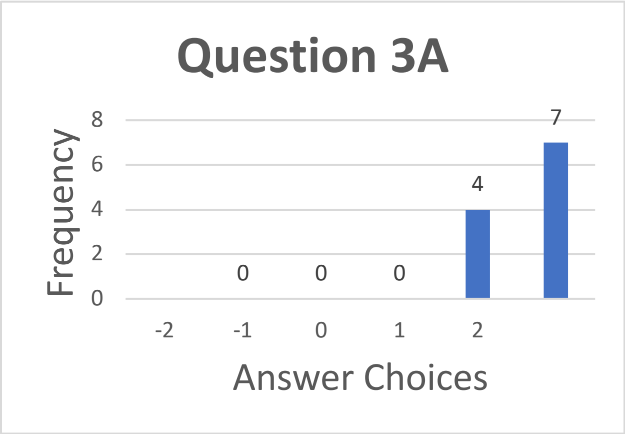
Question 3A
Frequency
8
6
4
2
0
7
4
0
0
0
-2
-1
0
1
2
Answer Choices

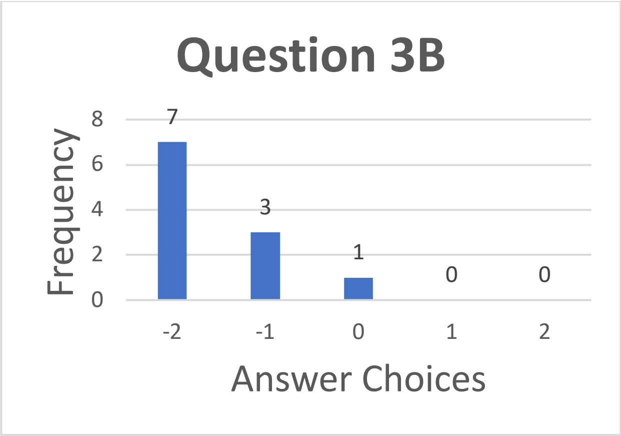
Question 3B
Frequency
8
6
4
2
0
7
3
1
0
0
-2
-1
0
1
2
Answer Choices

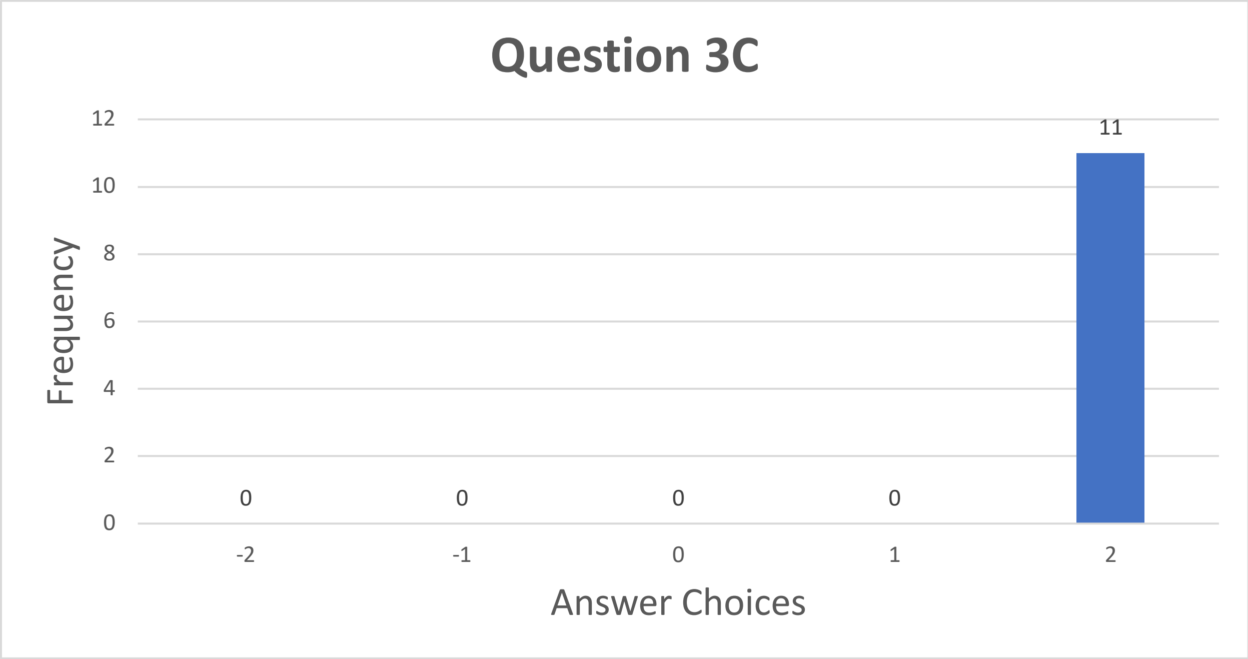
Question 3C
Frequency
12
10
8
6
4
2
0
11
0
0
0
0
-2
-1
0
1
2
Answer Choices

Rash

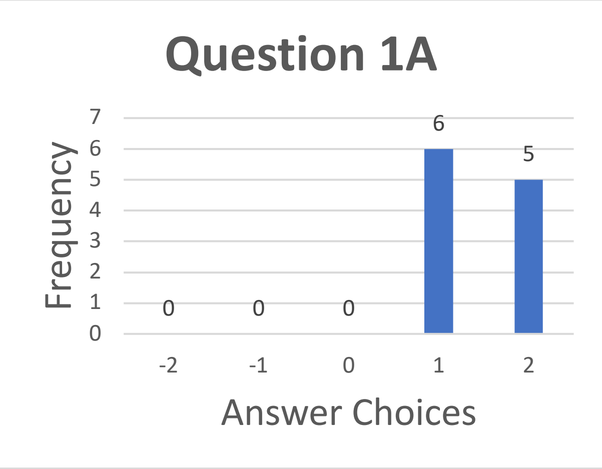
Question 1A
Frequency
Answer Choices
7
6
5
4
3
2
1
0
-2
-1
0
1
2
0
0
0
6
5

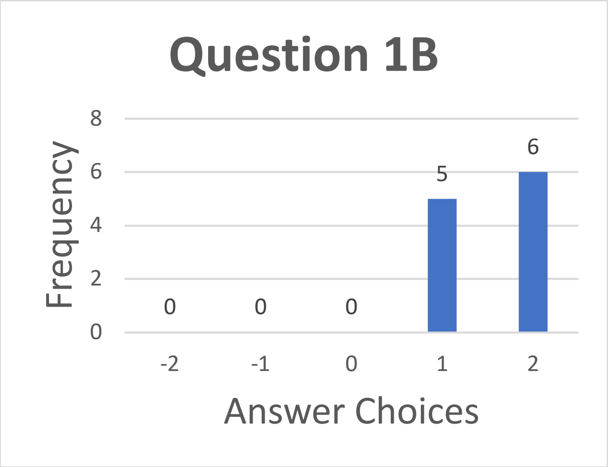
Question 1B
Frequency
Answer Choices
8
6
4
2
0
-2
-1
0
1
2
0
0
0
5
6

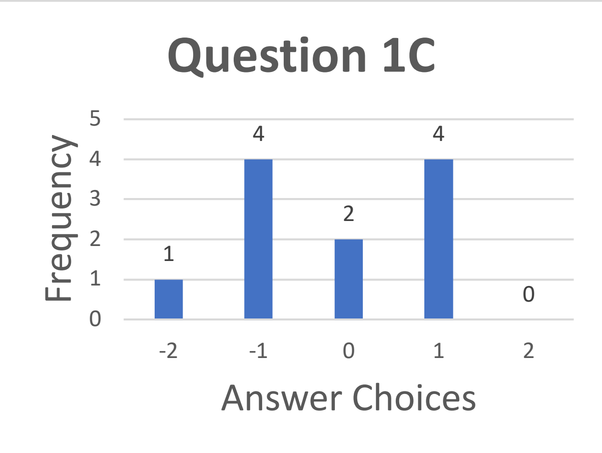
Question 1C
Frequency
Answer Choices
5
4
3
2
1
0
-2
-1
0
1
2
1
4
2
4
0

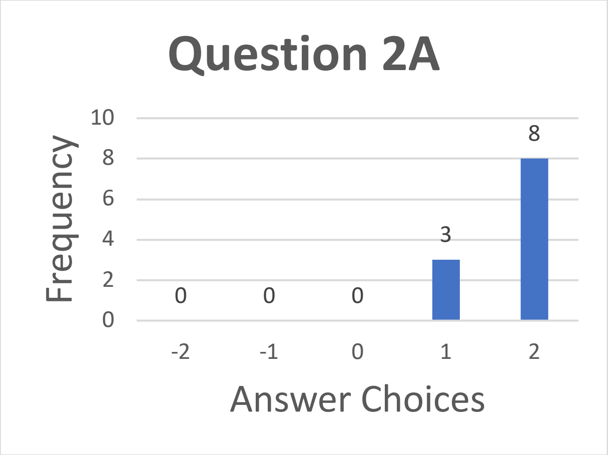
Question 2A
Frequency
Answer Choices
10
8
6
4
2
0
-2
-1
0
1
2
0
0
0
3
8

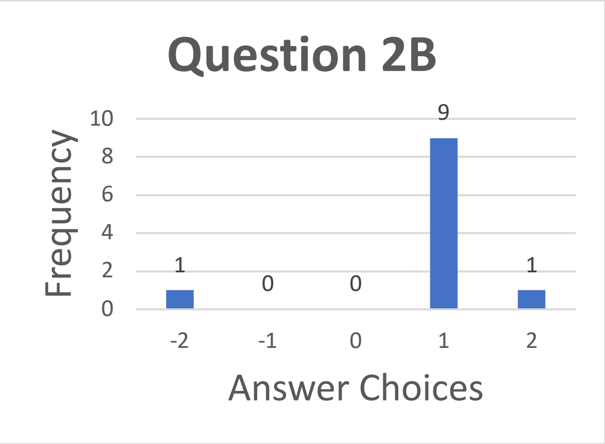
Question 2B
Frequency
Answer Choices
10
8
6
4
2
0
-2
-1
0
1
2
1
0
0
9
1

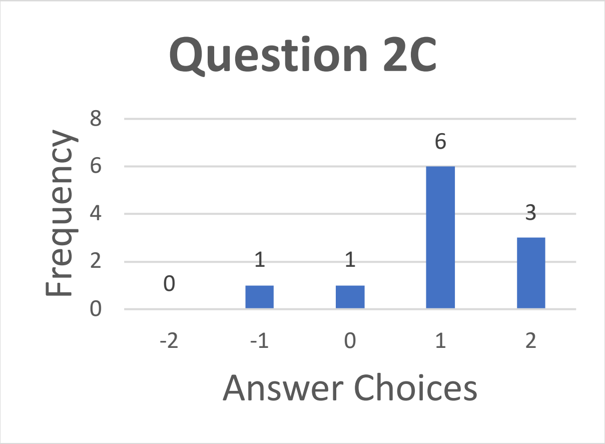
Question 2C
Frequency
Answer Choices
8
6
4
2
0
-2
-1
0
1
2
0
1
1
6
3

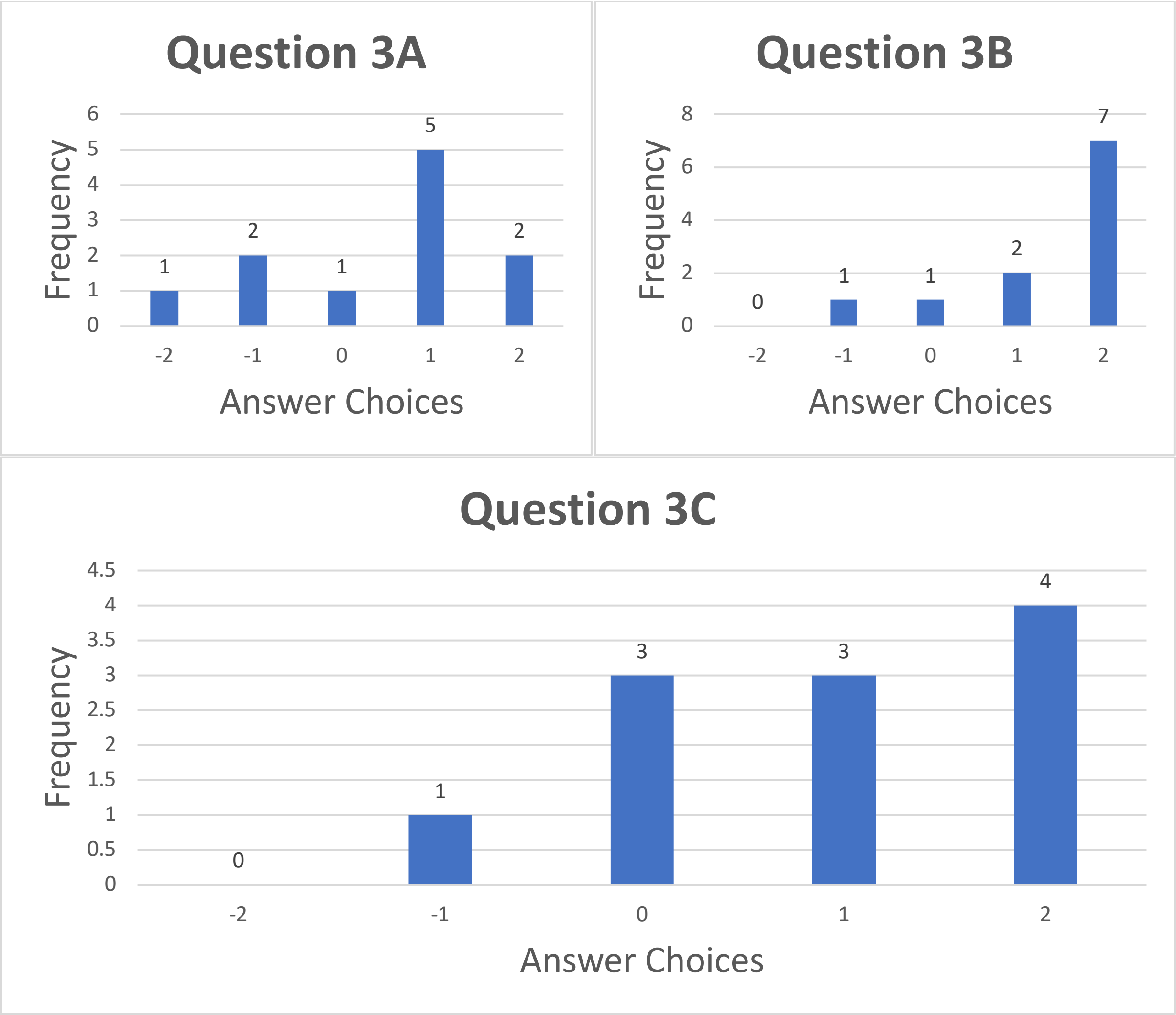

Question 3A
Frequency
6
5
4
3
2
1
0
1
2
1
5
2
-2
-1
0
1
2
Answer Choices
Question 3B
Frequency
8
6
4
2
0
0
1
1
2
7
-2
-1
0
1
2
Answer Choices
Question 3C
Frequency
4.5
4
3.5
3
2.5
2
1.5
1
0.5
0
0
1
3
3
4
-2
-1
0
1
2
Answer Choices

Abdominal Mass

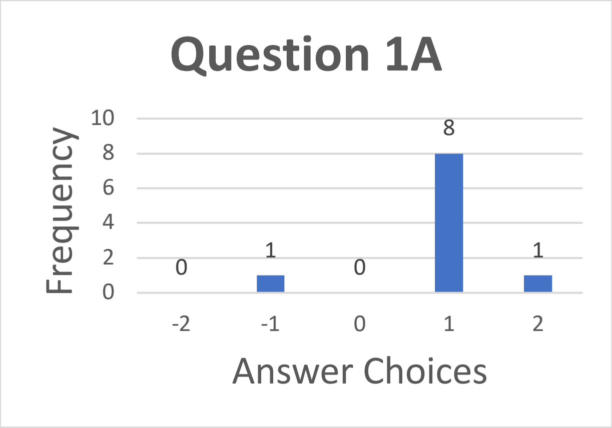

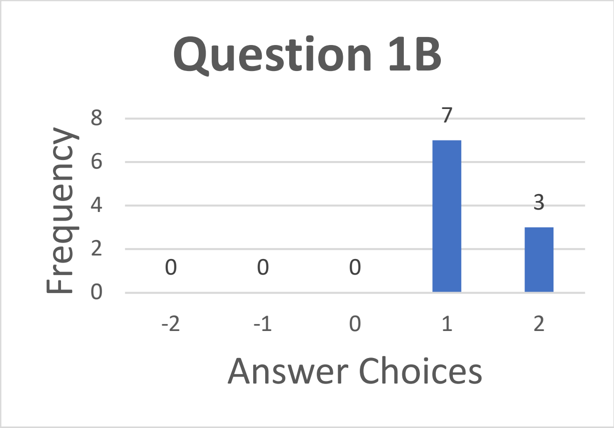

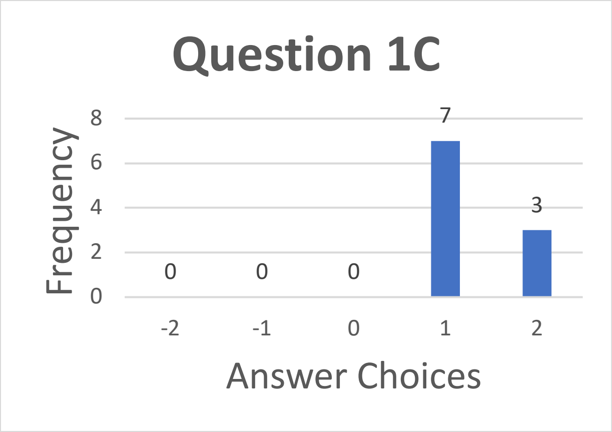

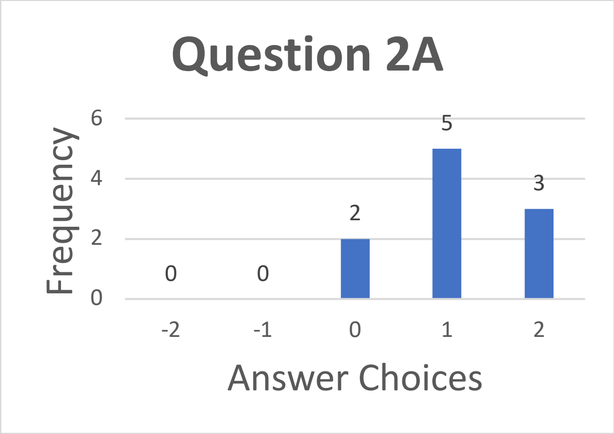

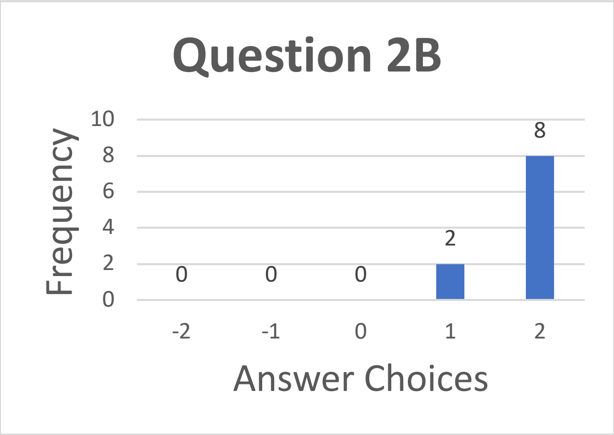

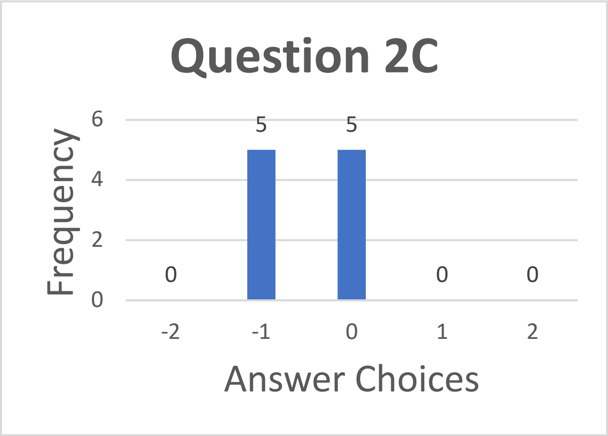

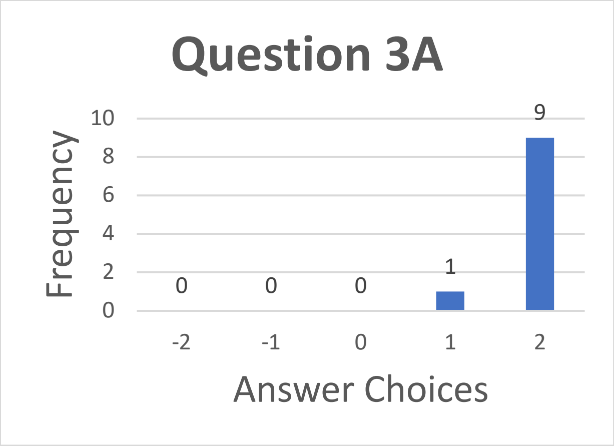
Question 3A
Frequency
10
8
6
4
2
0
0
0
0
1
9
-2
-1
0
1
2
Answer Choices

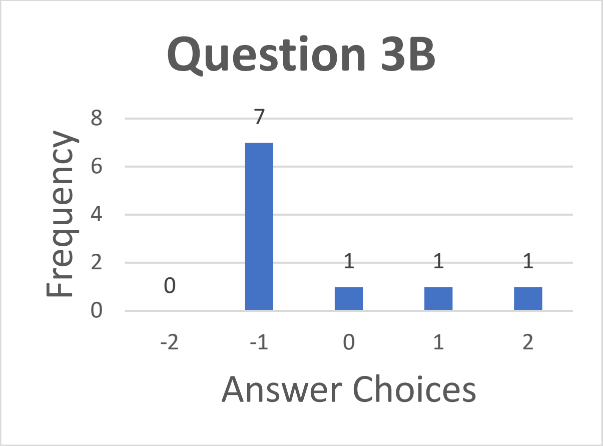
Question 3B
Frequency
8
6
4
2
0
0
7
1
1
1
-2
-1
0
1
2
Answer Choices

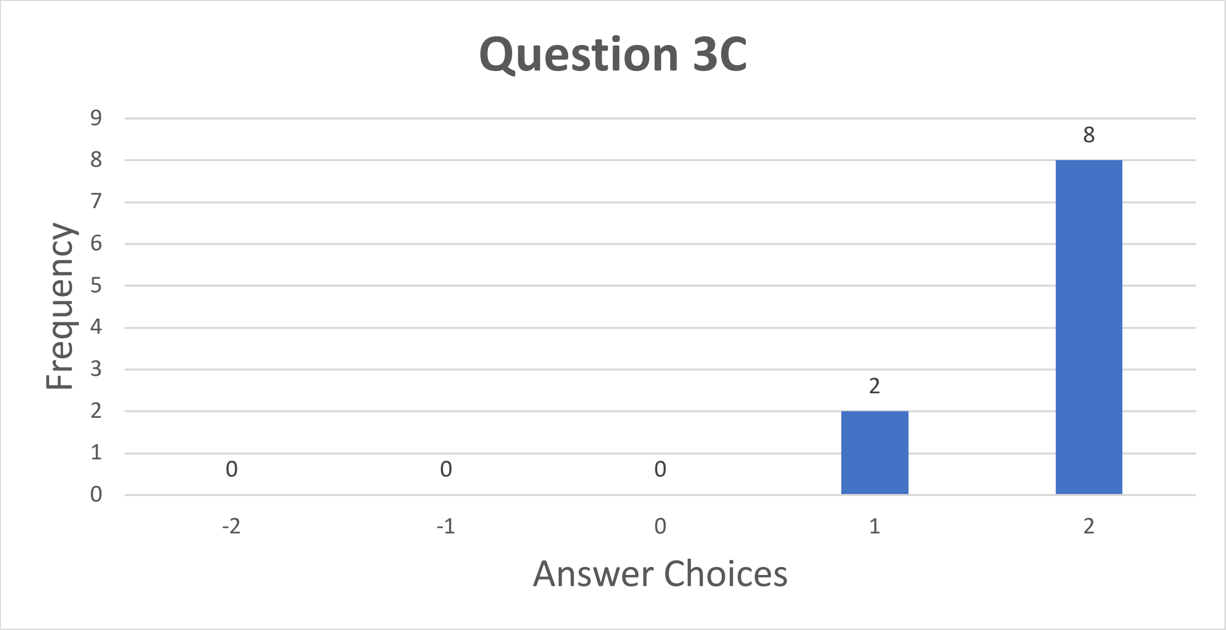
Question 3C
Frequency
9
8
7
6
5
4
3
2
1
0
0
0
0
2
8
-2
-1
0
1
2
Answer Choices

Diarrhea

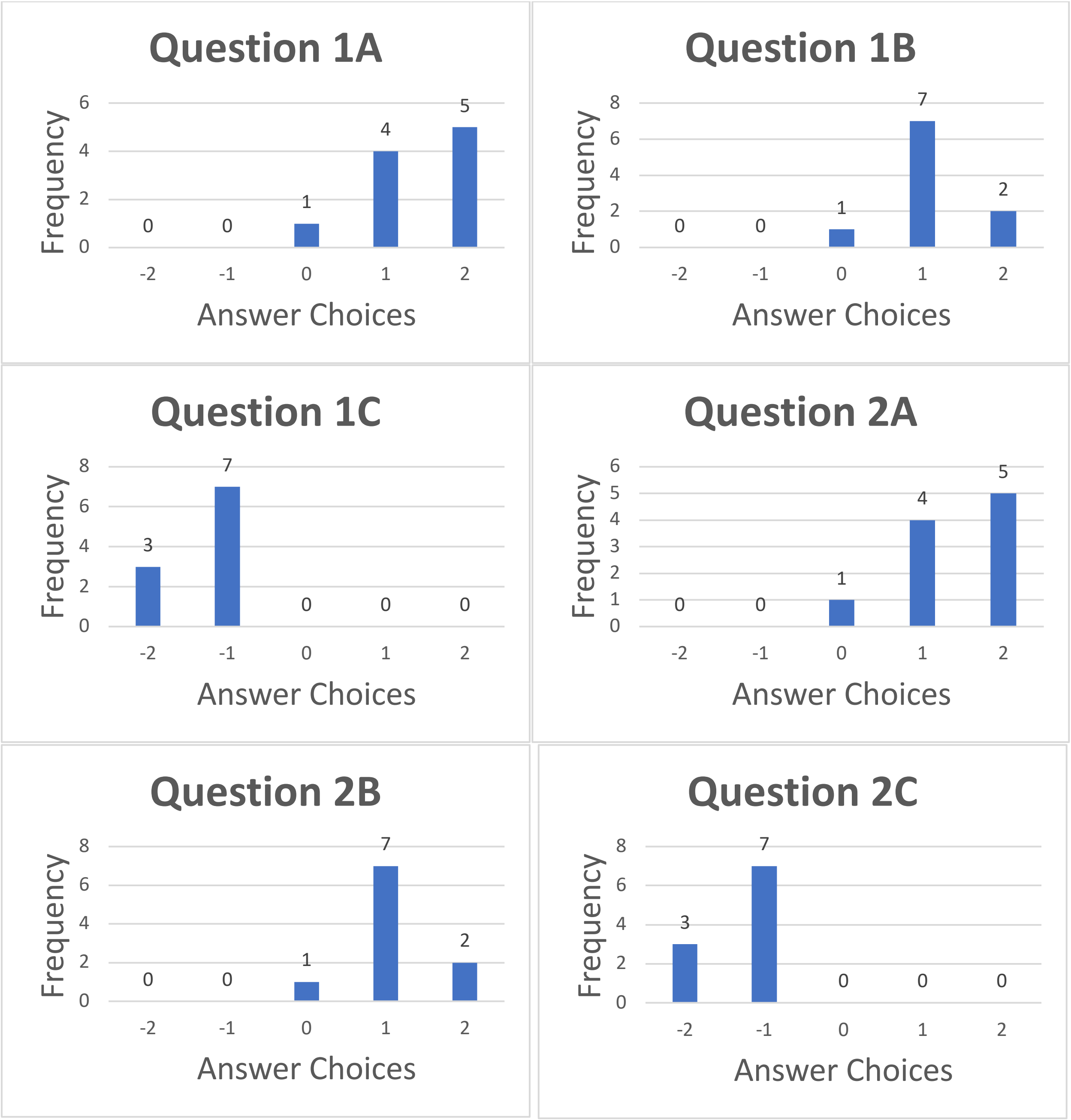

Question 1A
Frequency
6
4
2
0
0
0
1
4
5
-2
-1
0
1
2
Answer Choices
Question 1B
Frequency
8
6
4
2
0
0
0
1
7
2
-2
-1
0
1
2
Answer Choices
Question 1C
Frequency
8
6
4
2
0
3
7
0
0
0
-2
-1
0
1
2
Answer Choices
Question 2A
Frequency
6
5
4
3
2
1
0
0
0
1
4
5
-2
-1
0
1
2
Answer Choices
Question 2B
Frequency
8
6
4
2
0
0
0
1
7
2
-2
-1
0
1
2
Answer Choices
Question 2C
Frequency
8
6
4
2
0
3
7
0
0
0
-2
-1
0
1
2
Answer Choices

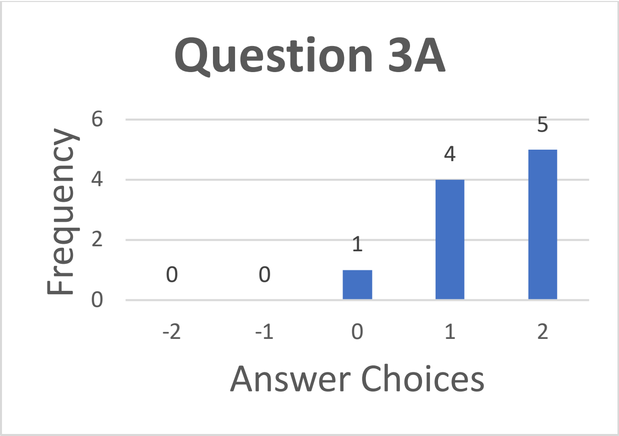
Question 3A
Frequency
Answer Choices
-2
-1
0
1
2
0
2
4
6
0
0
1
4
5

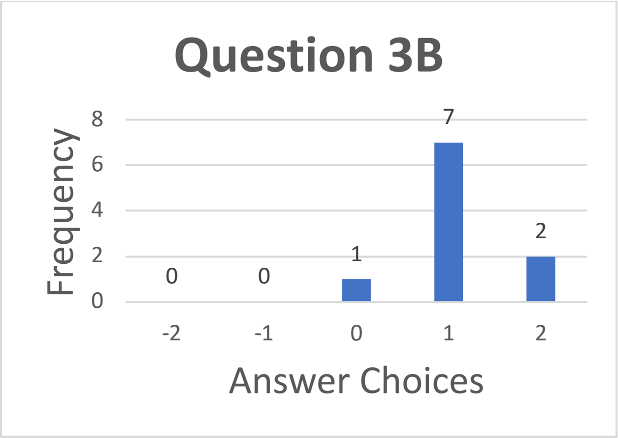
Question 3B
Frequency
Answer Choices
-2
-1
0
1
2
0
2
4
6
8
0
0
1
7
2

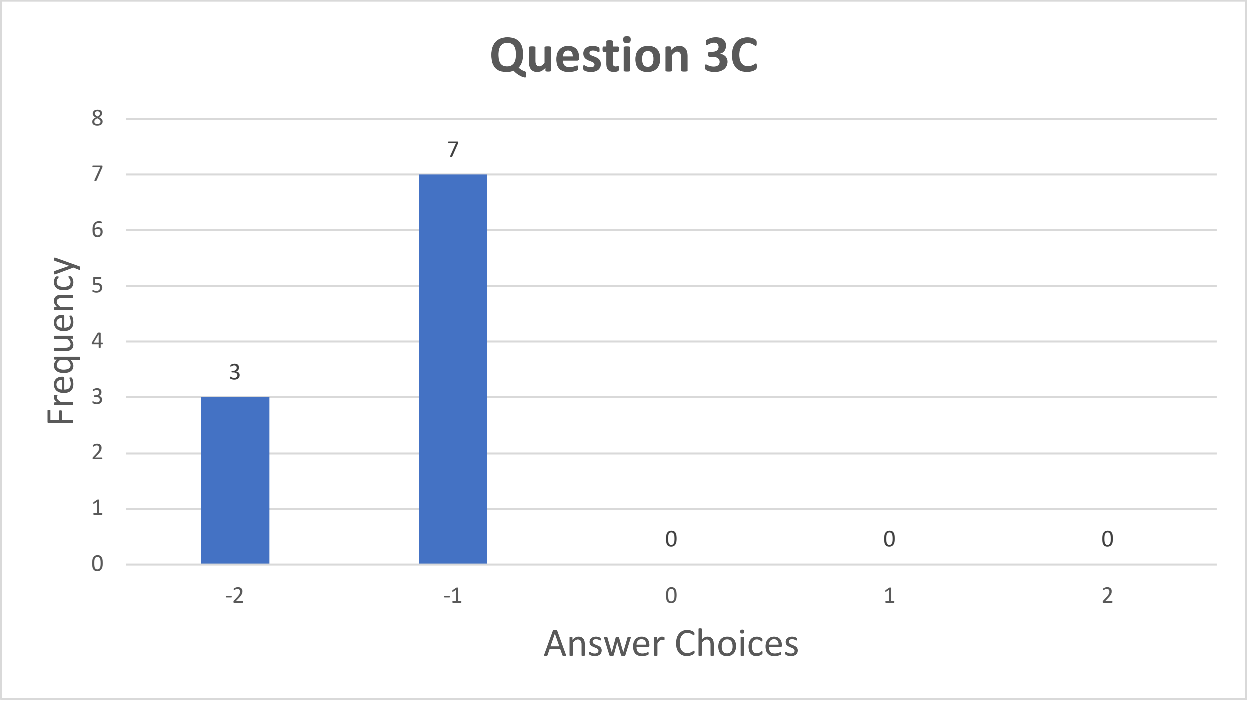
Question 3C
Frequency
Answer Choices
-2
-1
0
1
2
0
1
2
3
4
5
6
7
8
3
7
0
0
0

Lymphadenopathy

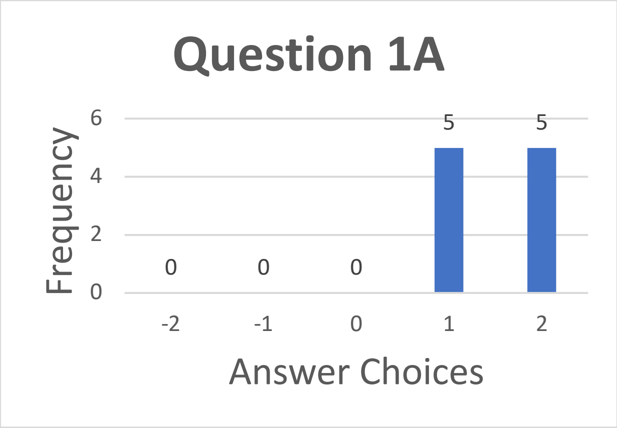

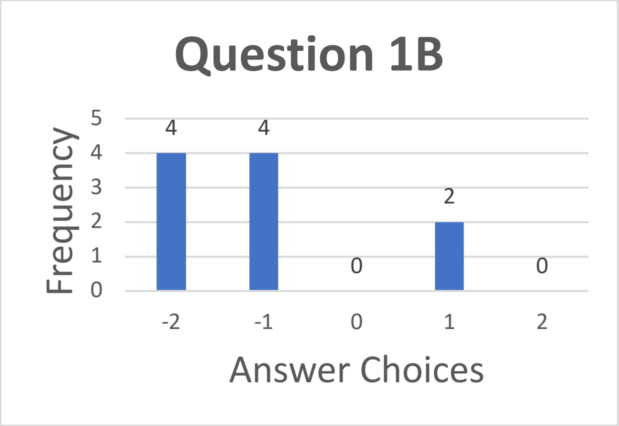

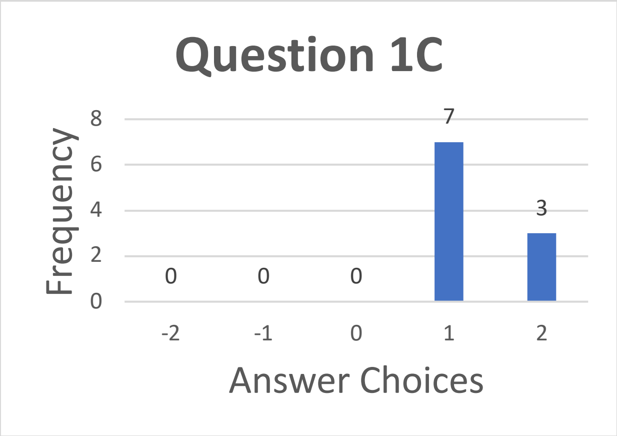

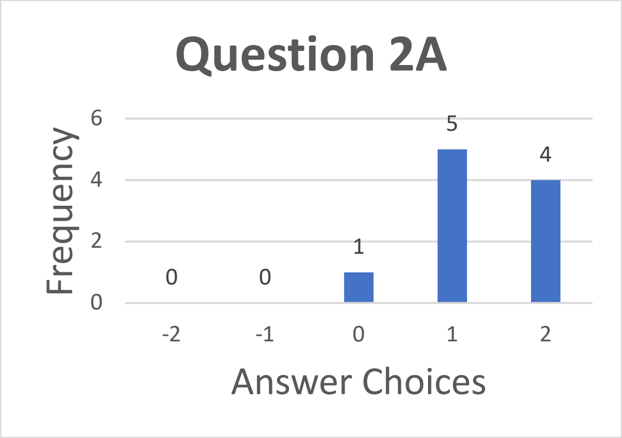

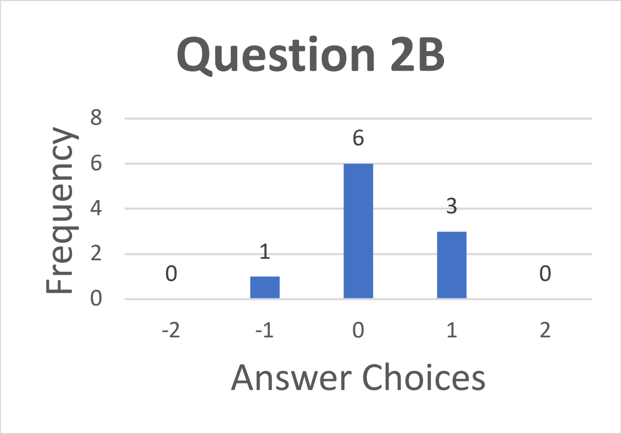

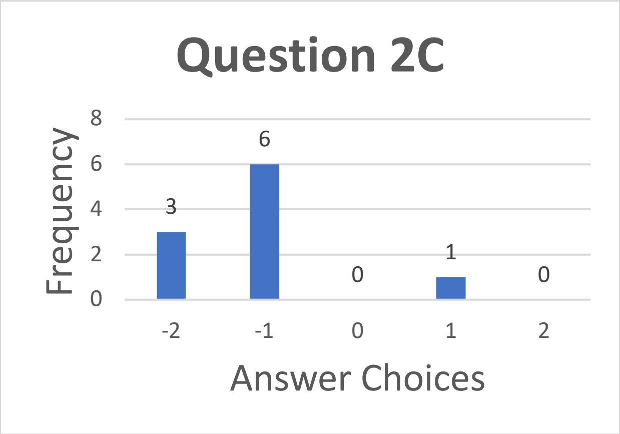

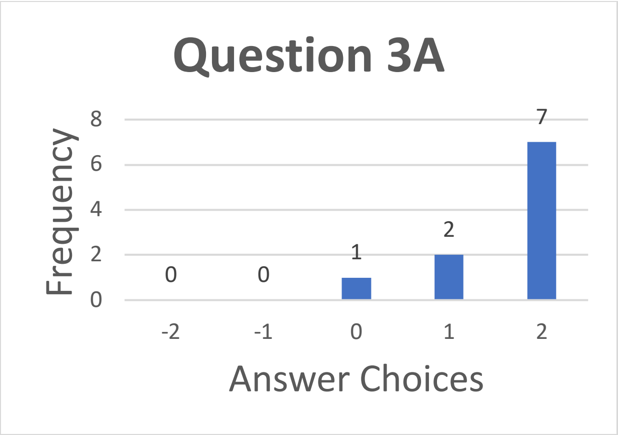
Question 3A
Frequency
8
6
4
2
0
0
0
1
2
7
-2
-1
0
1
2
Answer Choices

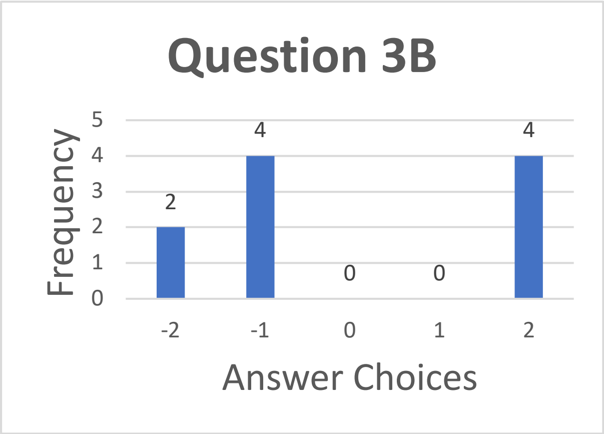
Question 3B
Frequency
5
4
3
2
1
0
2
4
0
0
4
-2
-1
0
1
2
Answer Choices

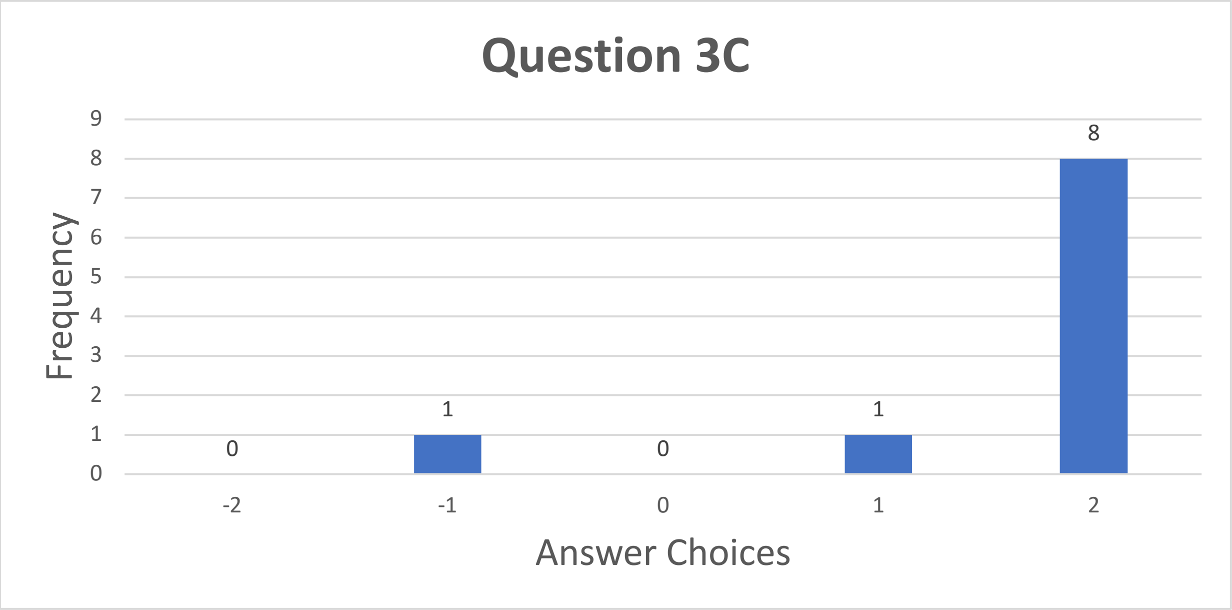
Question 3C
Frequency
9
8
7
6
5
4
3
2
1
0
0
1
0
1
8
-2
-1
0
1
2
Answer Choices

Otalgia

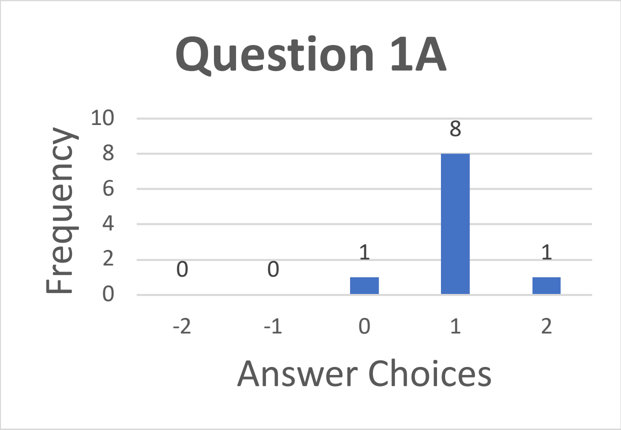
Question 1A
Frequency
10
8
6
4
2
0
0
0
1
8
1
-2
-1
0
1
2
Answer Choices

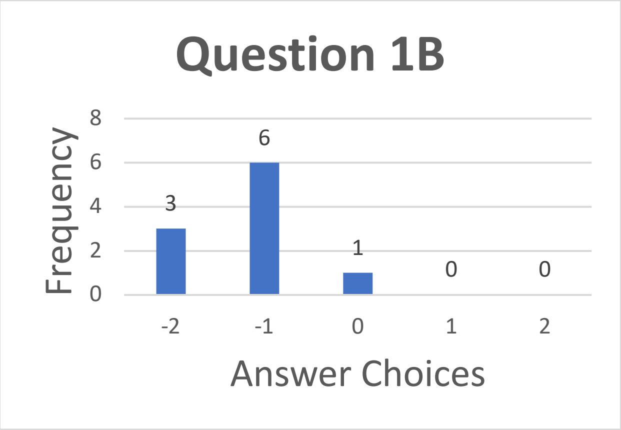
Question 1B
Frequency
8
6
4
2
0
3
6
1
0
0
-2
-1
0
1
2
Answer Choices

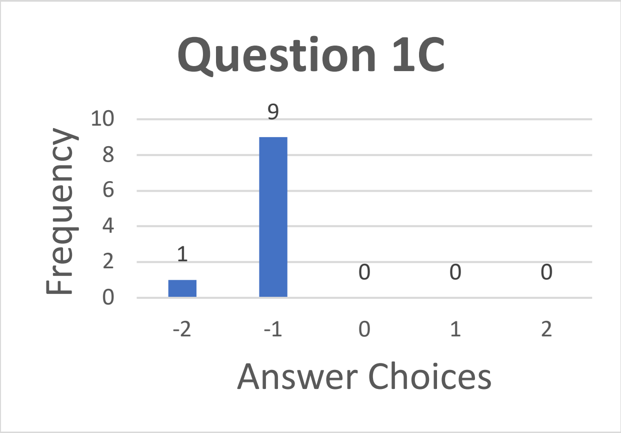
Question 1C
Frequency
10
8
6
4
2
0
1
9
0
0
0
-2
-1
0
1
2
Answer Choices

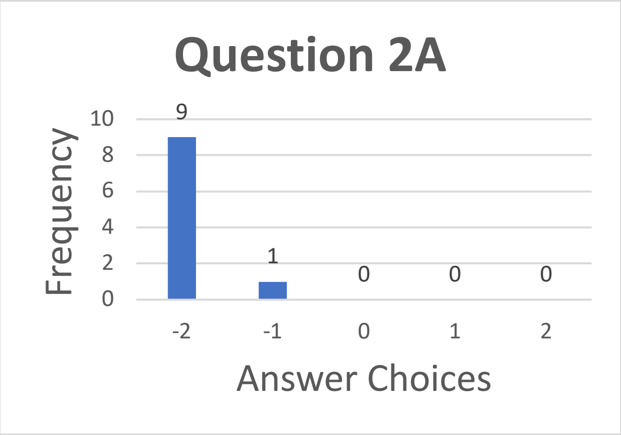
Question 2A
Frequency
10
8
6
4
2
0
9
1
0
0
0
-2
-1
0
1
2
Answer Choices

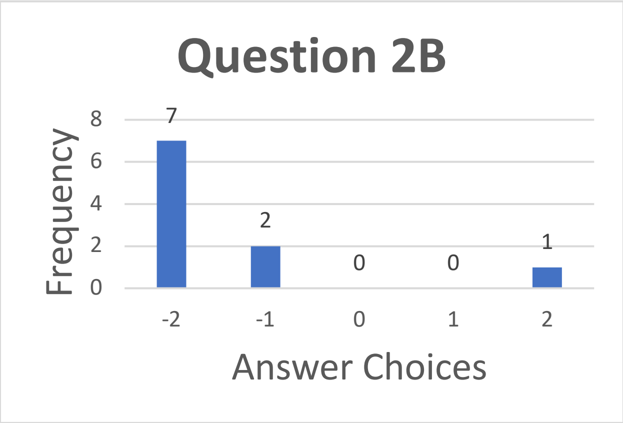
Question 2B
Frequency
8
6
4
2
0
7
2
0
0
1
-2
-1
0
1
2
Answer Choices

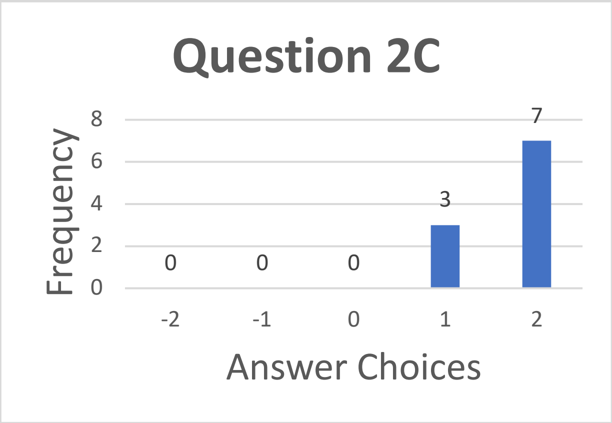
Question 2C
Frequency
8
6
4
2
0
0
0
0
3
7
-2
-1
0
1
2
Answer Choices

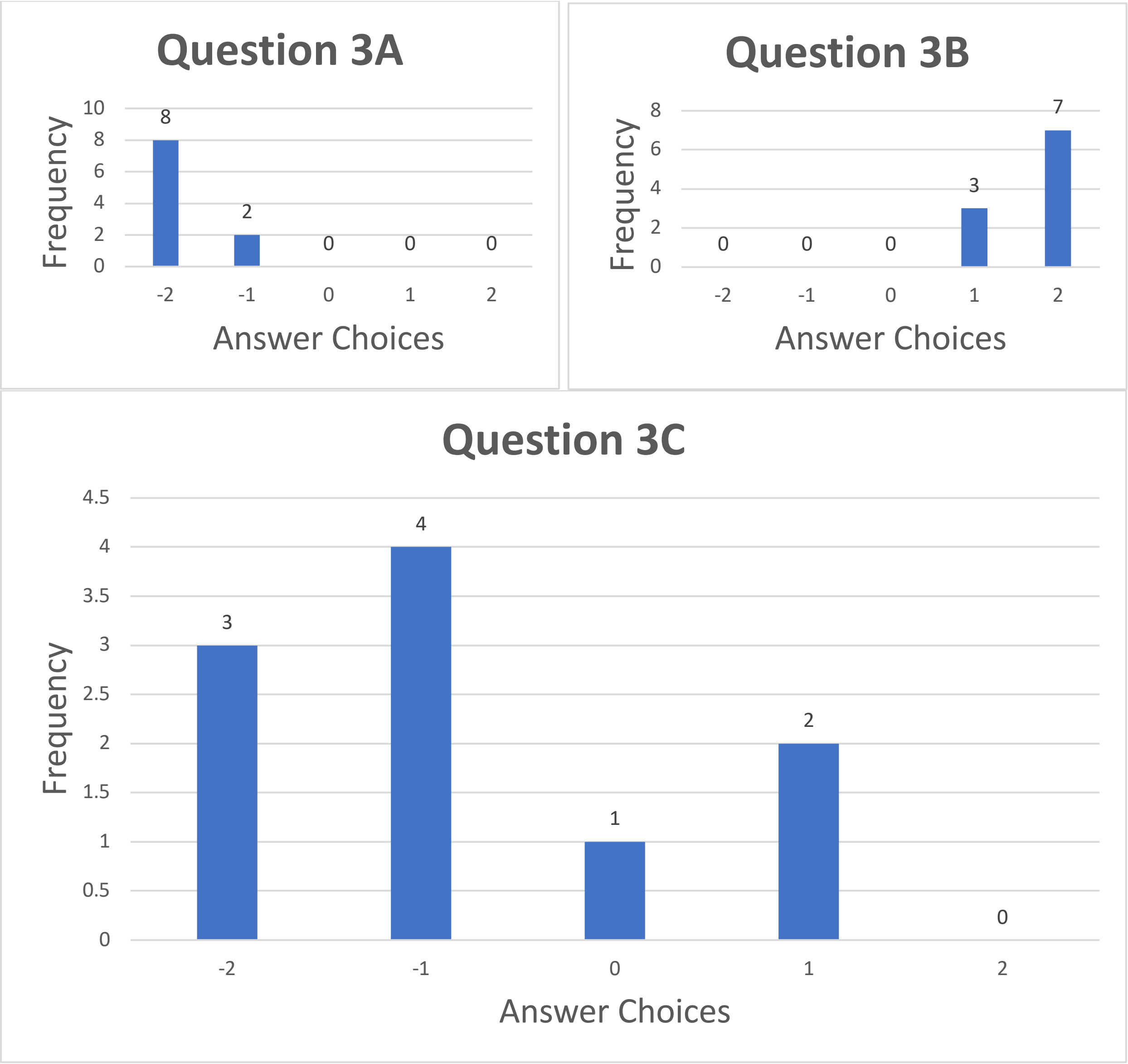
Question 3A
Frequency
10
8
6
4
2
0
8
2
0
0
0
-2
-1
0
1
2
Answer Choices
Question 3B
Frequency
8
6
4
2
0
0
0
0
3
7
-2
-1
0
1
2
Answer Choices
Question 3C
Frequency
4.5
4
3.5
3
2.5
2
1.5
1
0.5
0
3
4
1
2
0
-2
-1
0
1
2
Answer Choices

Vomiting

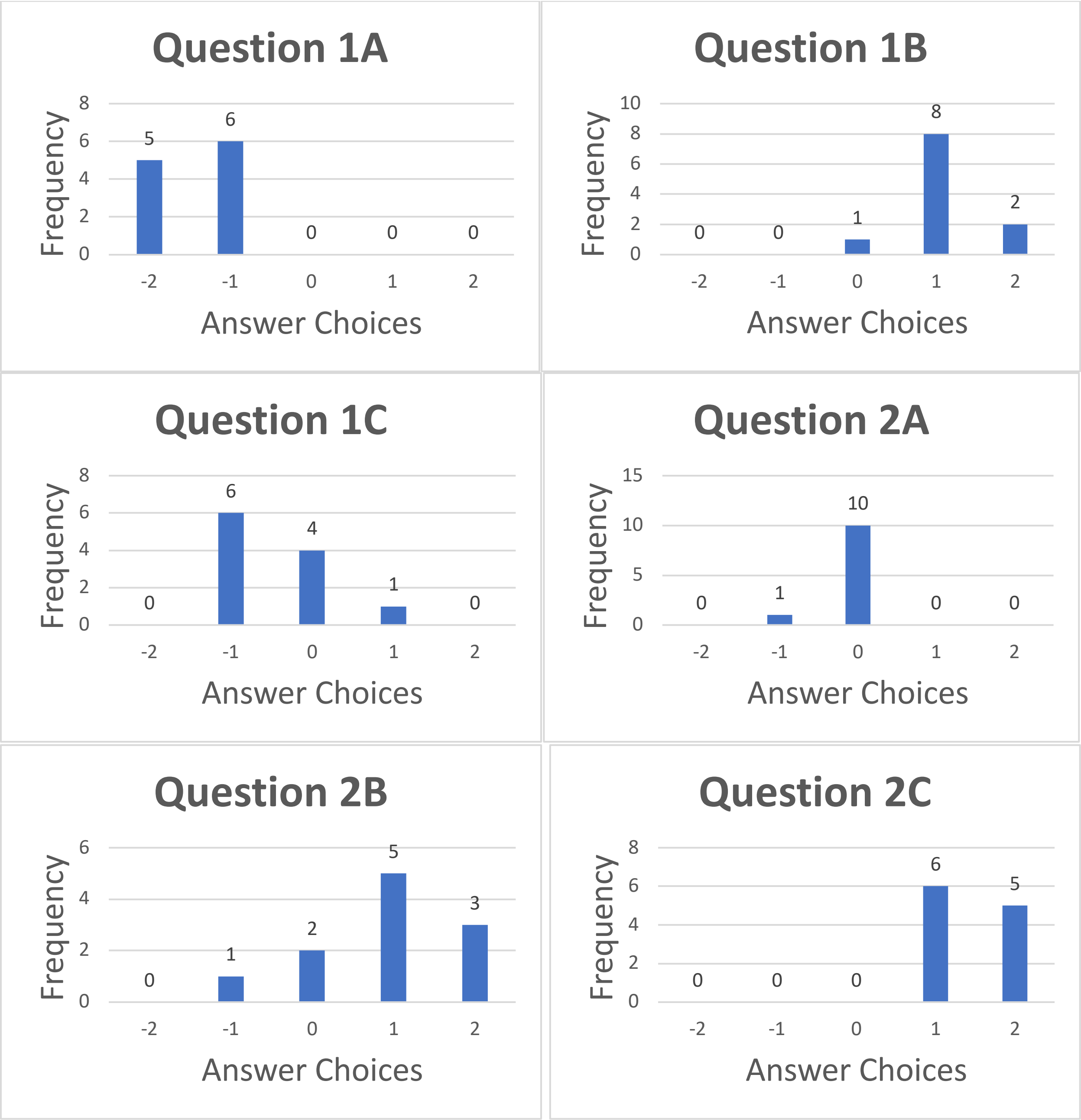

Question 1A
Frequency
8
6
4
2
0
5
6
0
0
0
-2
-1
0
1
2
Answer Choices
Question 1B
Frequency
10
8
6
4
2
0
0
0
1
8
2
-2
-1
0
1
2
Answer Choices
Question 1C
Frequency
8
6
4
2
0
0
6
4
1
0
-2
-1
0
1
2
Answer Choices
Question 2A
Frequency
15
10
5
0
0
1
10
0
0
-2
-1
0
1
2
Answer Choices
Question 2B
Frequency
6
4
2
0
0
1
2
5
3
-2
-1
0
1
2
Answer Choices
Question 2C
Frequency
8
6
4
2
0
0
0
0
6
5
-2
-1
0
1
2
Answer Choices

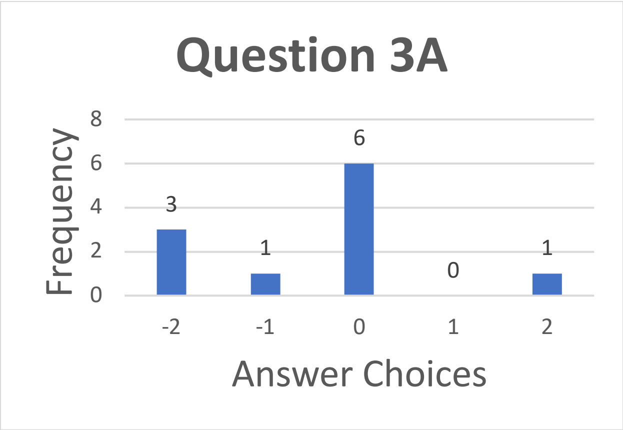
Question 3A
Frequency
0
2
4
6
8
3
1
6
0
1
-2
-1
0
1
2
Answer Choices

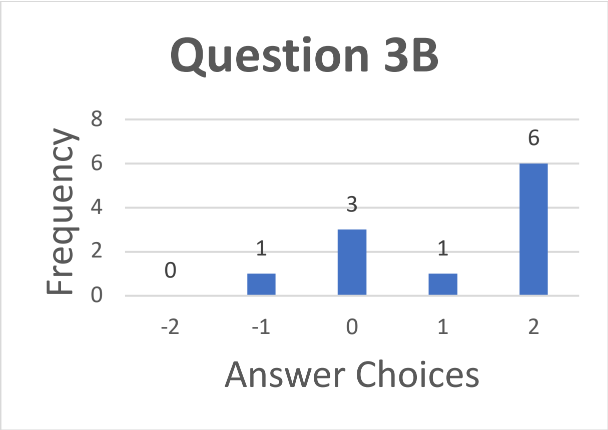
Question 3B
Frequency
0
2
4
6
8
0
1
3
1
6
-2
-1
0
1
2
Answer Choices

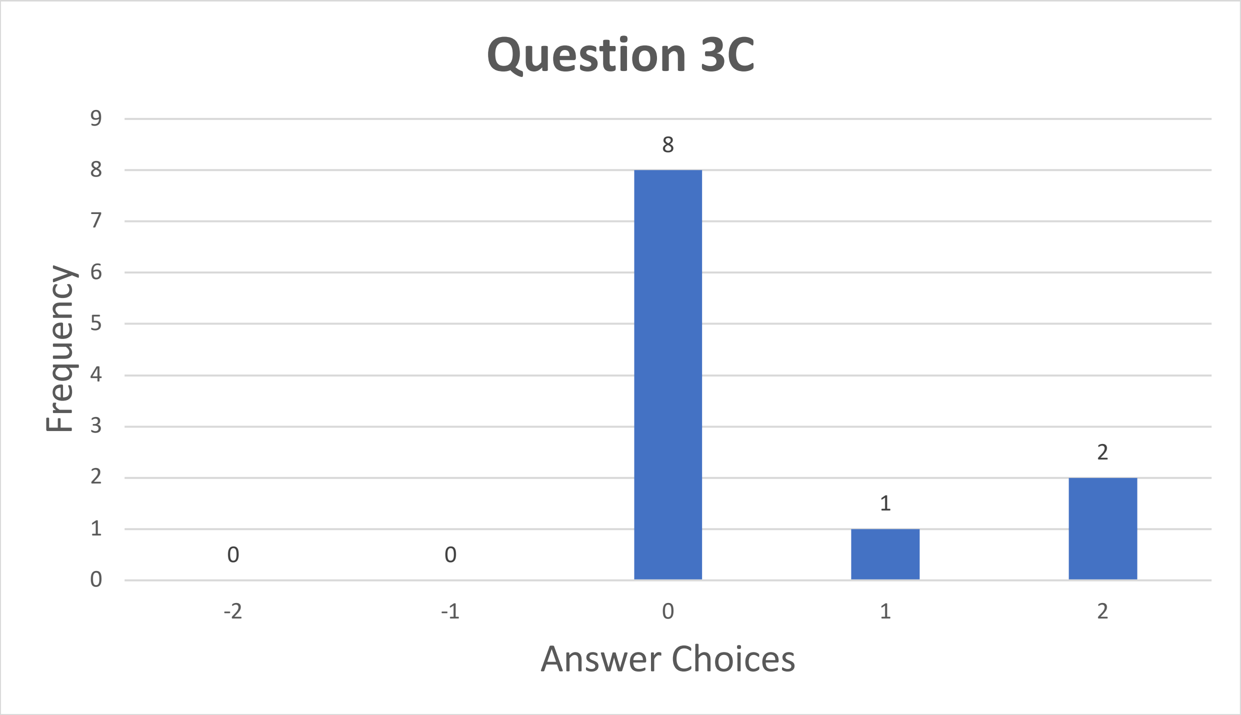
Question 3C
Frequency
0
1
2
3
4
5
6
7
8
9
0
0
8
1
2
-2
-1
0
1
2
Answer Choices

<u>Fever w/o a Source</u>

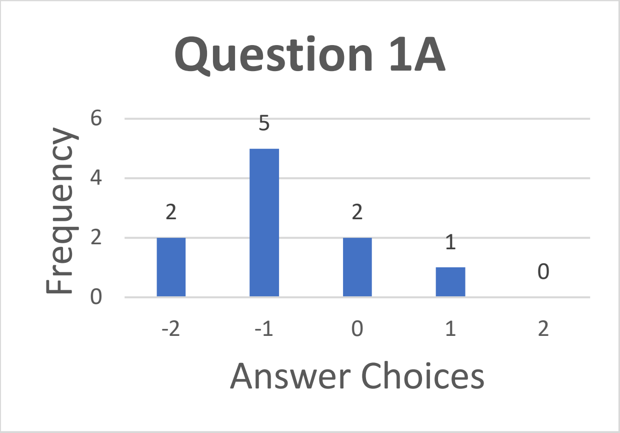

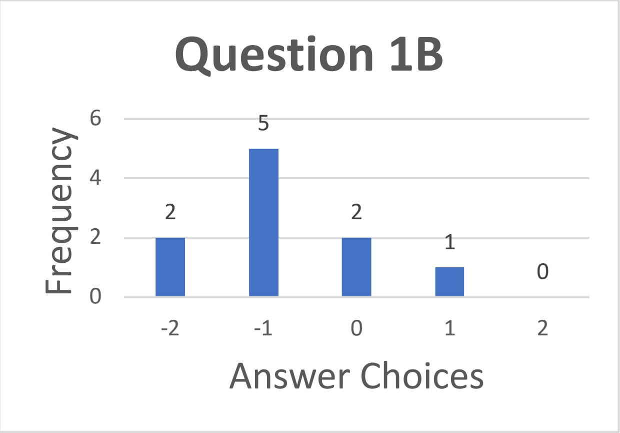

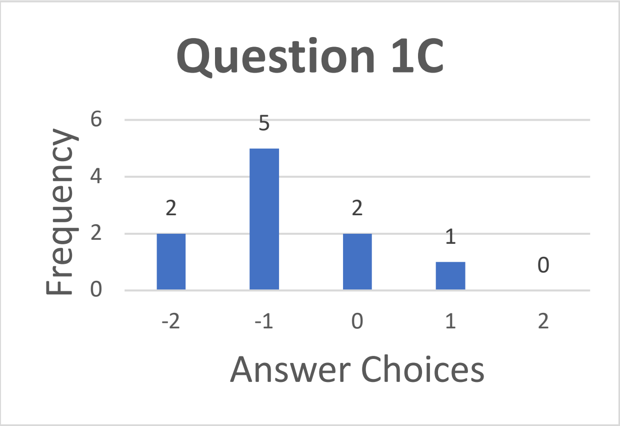

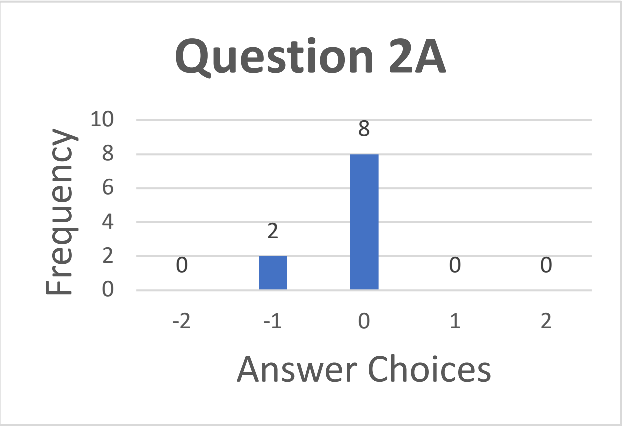

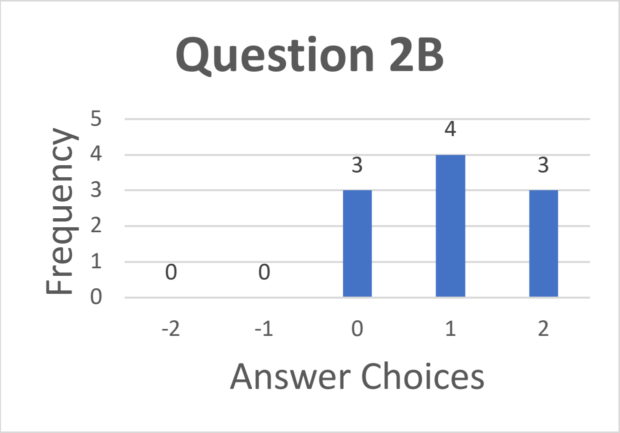

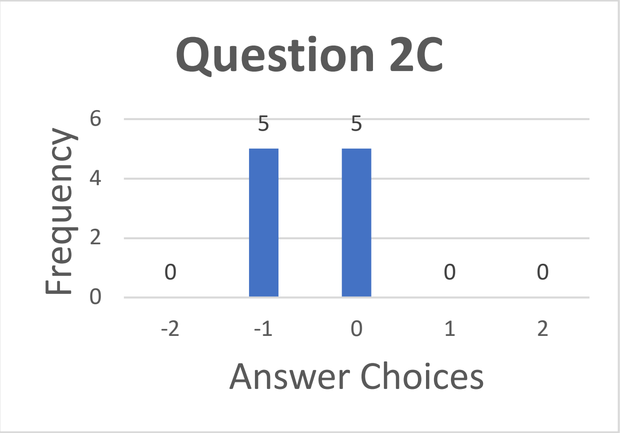

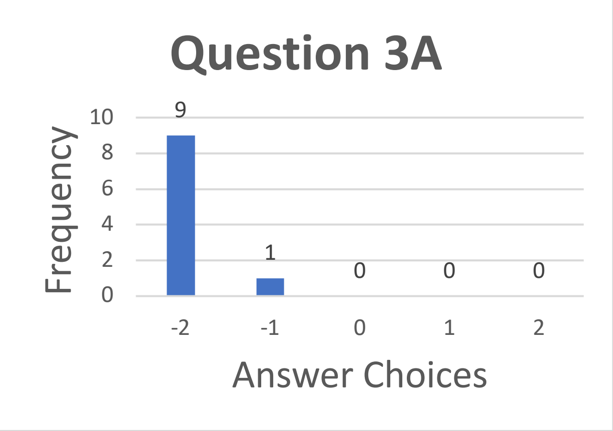
Question 3A
Frequency
10
8
6
4
2
0
9
1
0
0
0
-2
-1
0
1
2
Answer Choices

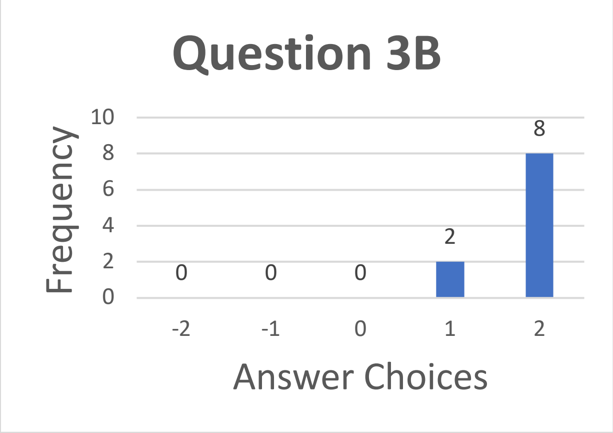
Question 3B
Frequency
10
8
6
4
2
0
0
0
0
2
8
-2
-1
0
1
2
Answer Choices

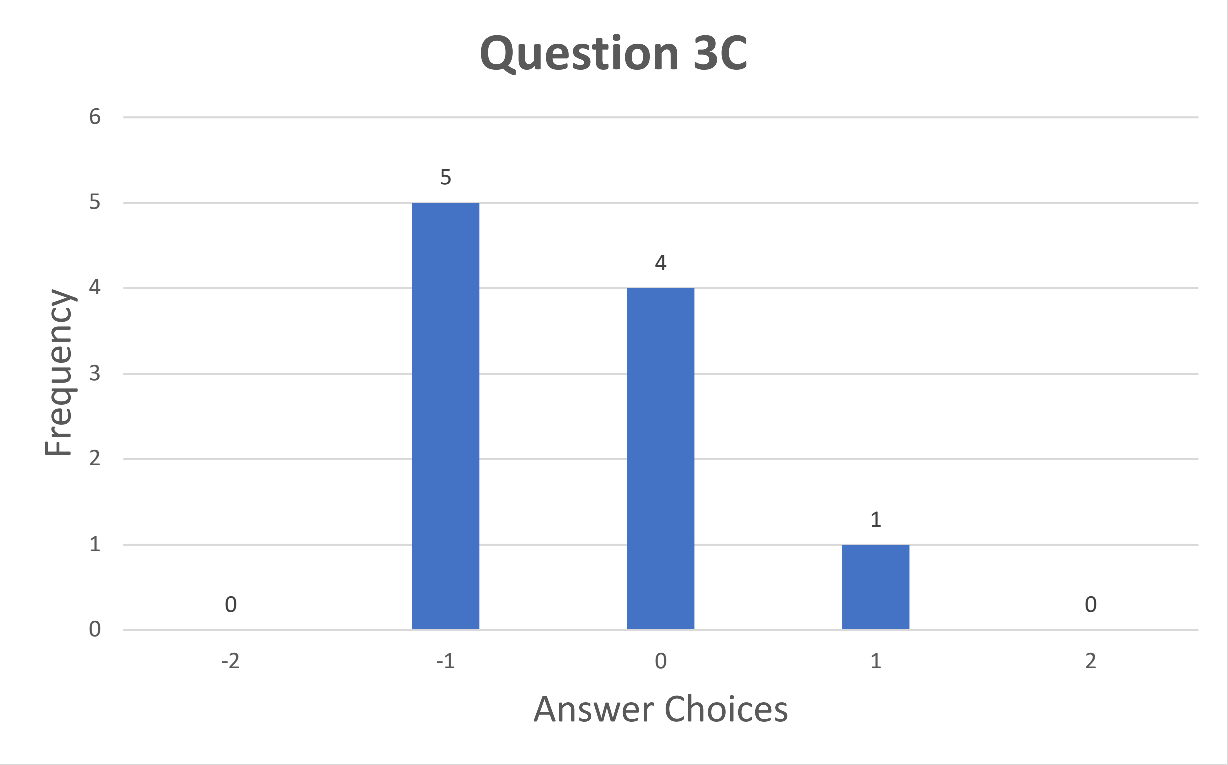
Question 3C
Frequency
6
5
4
3
2
1
0
0
5
4
1
0
-2
-1
0
1
2
Answer Choices
