# Supplementary material for: Script Concordance Tests for Formative Clinical Reasoning and Problem-Solving Assessment in General Pediatrics
Source: MedEdPORTAL. 2022 Sep 20;18:11274. doi: 10.15766/mep_2374-8265.11274 (PMC9485313; doi:10.15766/mep_2374-8265.11274)
Supplement: Supplementary file 1 — SCTs Without Answers.docxSCTs With Expert Answers.pdfScoring Guide.docxScoring Spreadsheet.xslx [file mep_2374-8265.11274-s001.zip › Educational_Summary_Report_11274.pdf]

MedEdPORTAL® The AAMC Journal of Teaching and Learning Resources

Original Publication Open Access

Script Concordance Tests for Formative Clinical Reasoning and Problem-Solving Assessment in General Pediatrics

Pranshu Bhardwaj*, Erik W. Black, PhD, MPH, Joseph C. Fantone III, MD, MS, Meghan Lopez, MS, Maria Kelly, MD

*Corresponding author: pbhardwaj@ufl.edu

Abstract

Introduction: Script Concordance Tests (SCTs) are short clinical vignettes with proposed diagnoses, diagnostic studies, treatments, and management options for patient care scenarios. The SCTs included in this resource were incorporated into a required pediatric clerkship to facilitate formative student feedback and additional opportunities for precepting faculty to provide midclerkship feedback. Pediatric cases were specifically selected due to the scarcity of medical student experience with common pediatric clinical presentations. **Methods:** We developed eight themed SCTs comprising 72 individual test items focused on common topics in general pediatrics. Items were administered to a convenience sample of third-year medical students during their required pediatric clerkship between fall 2016 and spring 2020. To evaluate the SCTs, we conducted item analyses, as well as comparing student performance to summative assessments. **Results:** The mean aggregate percentage score across all SCTs was .84 (*SD* = .08). Student SCT performance was related to USMLE Step 2 Clinical Knowledge scores, clerkship grades, and NBME Pediatrics Shelf Exam scores. **Discussion:** These SCTs facilitated feedback to medical students in the clinical learning environment. Their current form provides a means of exploring student clinical reasoning and problem-solving and can be used at a single point or to measure longitudinally. When paired with structured subject- and competency-specific midclerkship student evaluation, SCTs helped facilitate timely feedback to students via immediate explanations of each question. SCTs can assist students in recognizing and reflecting on potential knowledge gaps.

Keywords
Script Concordance Tests, Educational Assessments, Medical Decision-Making, Clinical Clerkship, Assessment, Clinical Reasoning/Diagnostic Reasoning, Pediatrics

Educational Objectives

By the using this assessment, facilitators will be able to:

1. Explore medical students' clinical reasoning and medical problem-solving using common pediatric clinical scenarios cases.
2. Incorporate objective measures of medical student performance during formative feedback.
3. Evaluate students' clinical reasoning and medical problem-solving growth over time.

Citation:
Bhardwaj P, Black EW, Fantone JC III, Lopez M, Kelly M. Script Concordance tests for formative clinical reasoning and problem-solving assessment in general pediatrics. *MedEdPORTAL*. 2022;18:11274. https://doi.org/10.15766/mep_2374-8265.11274

Introduction

Construct
Clinical reasoning and problem-solving are complex constructs that are challenging to assess systematically. These competencies are commonly assessed via clinical performance–based assessment by attending physicians during clinical rotations and written exams and in standardized patient encounters.[1]

Script Concordance Tests (SCTs), introduced by Charlin and colleagues,[2] have emerged as valid and reliable alternative to traditional multiple-choice questions for assessing student clinical competency.[3] SCTs consist of short clinical vignettes, typically with proposed diagnoses, diagnostic studies, treatments, and management options for patient care scenarios.[3] SCTs frequently guide the learner through an evolving clinical scenario, commonly comprising three or more parts. Charlin and colleagues described the fundamental components of a three-part SCT item[4]:

Copyright © 2022 Bhardwaj et al. This is an open-access publication distributed under the terms of the Creative Commons Attribution license.

- Part 1: diagnostic hypothesis, investigative action, or treatment option relevant to the situation.
- Part 2: new information (e.g., a sign, condition, imaging study, or laboratory test result) that might influence the diagnostic hypothesis, investigative action, or treatment option.
- Part 3: a Likert-type scale identifying how more or less likely a student is to make the diagnosis, order imaging/lab, or select a specific treatment based on the qualifier in part 2.

SCTs are constructed so that learners must answer each question independently before additional information is subsequently provided, requiring the examinees to determine the effect of that information on their decision regarding a diagnosis, test, or management option. In contrast to multiple-choice questions, SCTs evaluate a range of possible student responses to clinically ambiguous situations.[3] Well-constructed SCTs capture some of the ambiguity associated with a clinical encounter, recognizing that there may be no single best response to a scenario. The progression towards competency-based medical education has prompted investigation into types of formative assessment that can reliably and efficiently evaluate medical students in real time.[5] SCTs are not resource intensive but are suitable for online use and are reusable, making them a compelling option for medical educators.[2]

SCTs are a means of assessment that can provide faculty with objective information on learners' clinical and problem-solving competency. Prior work has described statistically significant positive relationships between SCT performance, clerkship clinical skill evaluations, and USMLE Step 2 Clinical Knowledge (CK) scores.[6-8] As more emphasis is placed upon medical student clerkship performance, objective measures for assessing learner skill and providing salient constructive feedback are needed. SCTs represent a promising form of assessment. Because SCTs evaluate judgment, they can be used to test clinical subjects where expert consensus on care delivery does not yet exist.[8] Cases remain the same, and the vignettes do not need to become more complex, as an increasing score on SCTs can be correlated to a subject's training level and track gains in clinical knowledge throughout an entire career.[3,8]

Intended Populations

These SCTs are intended for medical students and are best employed during a pediatric clerkship or subinternship. The items may also be useful for nonphysician health professions students (doctor of nursing practice and physician assistant).

Methods

Authors (Meghan Lopez and Joseph C. Fantone III) selected SCT topics and collaboratively wrote each associated vignette, representing eight common clinical scenarios in general pediatrics, including genetic syndromes, rashes, abdominal masses, diarrhea, lymphadenopathy, otalgia, vomiting, and fever without a source. Many of these topics were also covered in faculty taught in-house lectures during the pediatric clerkship. We have provided copies of each SCT to be administered to students and another copy with expert answers included (Appendices A and B, respectively). Vignettes were designed in a three-part scaffolded fashion; that is, the learner was provided information, prompted to respond to a set of questions, then offered additional information and another set of questions. Each SCT comprised nine items (72 individual test questions) divided equally among three categories:

1. The likelihood of a diagnosis based on historical or physical exam elements.
2. Evaluation of the relevance of ordering a lab or diagnostic study.
3. Evaluating the legitimacy of management strategies.

Following SCT construction, we administered the eight SCTs to an expert panel comprising 10 board-certified general pediatricians who responded to the items. The scoring key was developed by tabulating response frequencies at each point in the Likert-type response scale for each item.[9] For each question (72 total), each test (eight total), and aggregate AD scores over all items completed by each student, we adopted an absolute difference (AD) scoring method, using Bland and colleagues' 3-point absolute distance from the mean method for each separate SCT topic[9] (Appendices C and D). Mean aggregate scores were used to ensure comprehensive understanding of each clinical vignette.

The refined SCTs were administered during the required pediatric clerkship at the University of Florida College of Medicine to a convenience sample of third-year medical students from fall 2016 through spring 2020. Institutional review board approval was obtained prior to engaging in data analysis (UFIRB# 202000845).

Content Validity

During the item development process, we convened an expert panel of board-certified pediatricians who provided feedback on content validity. Each pediatrician had served in a faculty position at the University of Florida College of Medicine for an average of 13.4 years ($SD = 8.7$) of pediatric experience following completion of residency training. Thus, their

Copyright © 2022 Bhardwaj et al. This is an open-access publication distributed under the terms of the Creative Commons Attribution license.

experience in the field contributed to the content validity of our test items.

Results

We adopted the unitary theory of evidence, collecting validity data from varying sources to explore the utility of the SCTs.[10] The unitary theory of evidence seeks to comprehensively establish validity beyond simple calculations. We explored the validity of the SCTs via the lenses of construct, content, and consequential validity.

Between fall 2016 and summer 2020, 21 cohorts consisting of 455 students completed the required pediatric clerkship at the University of Florida College of Medicine. Students were instructed that the SCTs were part of a clinical reasoning exercise and, apart from one clerkship rotation, were optional. Clerkship materials, including the SCTs, were housed within the pediatric clerkship course residing in the Canvas Learning Management Students. All SCTs were made available to students at the start of the clerkship; each SCT was titled by subject matter (e.g., Clinical Reasoning Activity—Genetic Syndrome) and drew upon accumulative medical knowledge from the preclinical stages of the curriculum as well as learning materials and activities occurring in the early stages of the clerkship. Students could complete SCTs at any time during the clerkship. One-hundred thirty-one students (29%) completed at least one SCT. Eighteen (12%) of those respondents' answers were discarded because the test attempts were incomplete, resulting in a final sample of 113. Students who participated in the SCTs completed, on average, four of eight ($SD = 3.18$). The mean aggregate percentage AD score across all SCTs was .84 ($SD = .08$; Table 1). On average, students took 5.5 minutes ($SD = 1.1$) to complete an SCT. Thus, completing the entire set would take a student approximately 45 minutes. Faculty completed SCTs in an average of 3.5 minutes ($SD = 1.1$). Therefore, they took approximately 30 minutes complete the set.

We found the overall internal consistency using AD scoring to be .49 (95% CI, .42-.56). Pearson correlations comparing SCT performance to USMLE Step 1, NBME Pediatrics Shelf, NBME Pediatrics Shelf percentile, USMLE Step 2 CK, medical decision-making competency, and overall competency scores were all statistically significant at at least $p < .05$ (Table 2). When compared to Pearson correlations calculated using student NBME Shelf exam scores, SCTs demonstrated a slightly stronger but nonsignificant relationship to medical decision-making competency scores ($r = .267$ vs. $r = .265$, $p = .49$) and overall competency scores ($r = .237$ vs. $r = .192$, $p = .36$).

We evaluated the SCTs by exploring overall scores, internal consistency, and their relationship with summative assessments, including the USMLE Step 1 score, NBME Pediatrics Shelf Exam raw and percentiles score, USLME Step 2 CK score, clerkship medical decision-making competency score, and an overall clerkship competency score (measured on a 0-9 scale).

Construct validity was demonstrated by association between SCT scores and clinical evaluations of student medical decision-making by faculty throughout the rotation. Furthermore, increased performance of more experienced test takers (the pediatric attendings who also took the examination) indicates that higher levels of experience with pediatric clinical medicine correlated with better performance on the SCTs, further strengthening the construct validity of our assessment.

Clerkships have historically struggled to objectively query medical student clinical knowledge and reasoning in an efficient manner. Within the pediatric clerkship, following the completion of this analysis in fall 2020, the SCTs became a required assignment. SCT results are used to facilitate formative feedback during the midclerkship evaluation. Consequential validity was demonstrated in our case by leadership at the University of Florida discussing and encouraging wider adoption of SCTs in curriculum committee meetings after completion of our project. Leadership cited SCTs' potential to help guide midpoint formative feedback on student medical decision-making using an objective measure as the main motivation behind the push for universal and required SCT adoption.

Discussion

Exploring medical student decision-making and clinical reasoning in inherently ambiguous situations is an important yet challenging

Table 1. Script Concordance Test Descriptive Statistics

Statistic	Genetic Syndrome	Rash	Abdominal Mass	Diarrhea	Lymphadenopathy	Otalgia	Vomiting	Fever Without a Source
N	94	75	64	62	57	53	53	52
Average	.91	.79	.88	.86	.81	.78	.82	.75
SD	.07	.10	.11	.09	.10	.14	.11	.14
Variance	.00	.01	.01	.01	.01	.02	.01	.02
Median	.94	.81	.91	.88	.84	.77	.82	.77

Copyright © 2022 Bhardwaj et al. This is an open-access publication distributed under the terms of the Creative Commons Attribution license.

Table 2. Pearson Correlation Coefficients Between Aggregate SCT Score and Outcomes Measures

Category	Step 1	Pediatric Shelf Score	Pediatric Shelf Percentile Score	Step 2 CK	Medical Decision-Making Competency	Competency Score
Cumulative SCT score	.22[a]	.30[c]	.31[c]	.23[a]	.27[b]	.24[a]
95% CI	.04-.39	.12-.46	.10-.47	.04-.41	.09-.43	.05-.40

Abbreviations: CK, clinical knowledge; SCT, Script Concordance Test.
[a] $p \leq .05$.
[b] $p \leq .01$.
[c] $p \leq .001$.

endeavor. SCTs provide an economical alternative to multiple-choice questions for querying learner skills. Multiple studies have explored the validity of SCTs and advocated for their incorporation into medical curricula.[2,4,8,9,11,12] These pediatric SCTs were well suited for use in our pediatric clerkship. They demonstrated positive relationships with summative measures of consequence (NBME Pediatrics Shelf Exam, USMLE Step 2 CK, and competency scores); item properties and interest in wider institutional adoption provide evidence of validity and utility. While we are not asserting generalizability across settings, our results are congruent with Humbert and colleagues' exploration of SCTs and their relationship to summative measures in emergency medicine trainees.[8]

Based on our experience, we feel that SCTs are well suited for facilitating meaningful conversations with learners, but additional work is needed before we employ them as more consequential forms of assessment. SCTs can be reused without concern for item deterioration, providing a promising means of assessing competency growth over time, identifying gaps in performance, and evaluating the need for and appraising the progression of remediation.[13] These unique qualities make SCTs promising facilitators for specific, low-cost, timely, midclerkship feedback to learners. Students received feedback via real-time explanation of each individual question by the test writers. Furthermore, SCTs were strongly suggested to students who had shown gaps in clinical reasoning knowledge in previous clerkships. Our overall analysis of these data in totality took place following completion of the clerkships by these medical students. As a result, clerkship directors were unable to use our analysis for formative feedback of students. However, as mentioned above, students could compare their responses to faculty experts in real time as a form of immediate feedback on their performance. This statement was included in our write-up because it has been well documented that SCT scores positively correlate with increased levels of training and application of knowledge.

Midclerkship feedback is a Liaison Committee on Medical Education requirement for clerkships longer than 4 weeks.[14] We hope to implement SCTs formally in our midpoint formative feedback of students as an example of the clinical reasoning competency. To do this, we will need to make SCTs required.

Finally, we postulate that in the future, with continued research and development, SCTs could be used as an evaluative measure of the quality of clinical education and the clinical learning environments associated with a clerkship. For example, a clerkship director at a medical school with multiple rotation sites could incorporate SCT performance data from each site to better inform needs for specific faculty or site development. Markert and colleagues offered evidence for a similar method of comparing the quality of clinical site instruction by contrasting student NBME scores and clerkship grades, along with other postgraduate measures.[15]

Limitations

These SCTs have notable limitations. Their use was limited to a single institution, with a limited sample size. Our exploration did not control for cohort maturation effects during the academic years, nor was student demographic information incorporated. SCTs were optional for most clerkship students, resulting in the possibility of selection bias. Furthermore, time between SCT completion and Step 2 CK completion was widely variable between students due to the varying order of third-year clerkships, which could be considered a confounding variable. In addition, SCT internal consistency was lower than desirable (.49). To achieve a Cronbach alpha value of .65, the number of cases would need to be increased from eight to 16, assuming similar difficulty, which is in line with recommendations by Gagnon and colleagues.[11] However, we do not perceive a high Cronbach alpha value as a requirement for meaningful implementation of our learning tool, given that this is a formative assessment with no bearing on students' grades. Correlation coefficients between SCTs and summative assessments were lower than anticipated but were in line with the range of mini-Clinical Evaluation Exercise data reported by Mortaz Hejri and colleagues.[16]
We feel that a natural next step would involve collaborative multi-institutional SCT item development, implementation, and exploration.

Copyright © 2022 Bhardwaj et al. This is an open-access publication distributed under the terms of the Creative Commons Attribution license.

Conclusion

SCTs administered during a pediatric clerkship were positively related to summative outcome measures including NBME performance, Step 2 CK scores, medical decision-making clerkship competency score, and overall clerkship competency score. Based upon this information, future University of Florida College of Medicine pediatric clerkship students will be required to complete SCTs, and additional SCTs will be developed to improve internal consistency. SCTs will continue to be used to facilitate robust midclerkship feedback. Additional work with regard to standardization of SCT grading, scaling, and student familiarity with the format is necessary before we can consider the use of SCTs as summative assessments. Further projects could feasibly explore the relationship between SCTs, summative outcomes, and student demographics, as well as the potential for SCTs as formative and summative assessment tools.

Appendices

A. SCTs Without Answers.docx

B. SCTs With Expert Answers.pdf

C. Scoring Guide.docx

D. Scoring Spreadsheet.xslx

All appendices are peer reviewed as integral parts of the Original Publication.

Pranshu Bhardwaj: Third-Year Medical Student, University of Florida College of Medicine; ORCID: https://orcid.org/0000-0003-2104-2085

Erik W. Black, PhD, MPH: Associate Professor, Department of Pediatrics, University of Florida College of Medicine

Joseph C. Fantone III, MD, MS: Senior Associate Dean for Educational Affairs and Professor, Department of Pathology, University of Florida College of Medicine

Meghan Lopez, MS: Assistant Director, Education and Training Programs, Department of Pediatrics, University of Florida College of Medicine

Maria Kelly, MD: Director of Medical Student Education and Professor, Department of Pediatrics, University of Florida College of Medicine

Acknowledgments

The authors would like to thank and acknowledge Dr. Lou Ann Cooper's role in data acquisition for this study.

Disclosures

None to report.

Funding/Support

None to report.

Prior Presentations

Bhardwaj P, Black EW, Fantone JC III, Lopez M, Kelly M. Exploring script concordance data as a formative assessment tool in a pediatrics clerkship. Poster presented virtually at: Pediatric Academic Societies Annual Meeting; April 30-May 4, 2021.

Bhardwaj P, Black EW, Fantone JC III, Lopez M, Kelly M. Exploring script concordance data as a formative assessment tool in a pediatrics clerkship. Presented virtually at: Medical Student Research Day, University of Florida College of Medicine; February 16, 2021.

Ethical Approval

The University of Florida Institutional Review Board deemed further review of this project not necessary.

References

1. Kreiter CD, Bergus G. The validity of performance-based measures of clinical reasoning and alternative approaches. *Med Educ*. 2009;43(4):320-325. https://doi.org/10.1111/j.1365-2923.2008.03281.x
2. Charlin B, Roy L, Brailovsky C, Goulet F, van der Vleuten C. The Script Concordance Test: a tool to assess the reflective clinician. *Teach Learn Med*. 2000;12(4):189-195. https://doi.org/10.1207/S15328015TLM1204_5
3. Humbert AJ, Miech EJ. Measuring gains in the clinical reasoning of medical students: longitudinal results from a school-wide Script Concordance Test. *Acad Med*. 2014;89(7):1046-1050. https://doi.org/10.1097/ACM.0000000000000267
4. Charlin B, Tardif J, Boshuizen HPA. Scripts and medical diagnostic knowledge: theory and applications for clinical reasoning instruction and research. *Acad Med*. 2000;75(2): 182-190. https://doi.org/10.1097/00001888-200002000-00020
5. Lievens F. Adjusting medical school admission: assessing interpersonal skills using situational judgement tests. *Med Educ*. 2013;47(2):182-189. https://doi.org/10.1111/medu.12089
6. Kelly W, Durning S, Denton G. Comparing a script concordance examination to a multiple-choice examination on a core internal medicine clerkship. *Teach Learn Med*. 2012;24(3):187-193. https://doi.org/10.1080/10401334.2012.692239
7. Rosenthal S, Russo S, Berg K, et al. Identifying students at risk of failing the USMLE Step 2 Clinical Skills examination. *Fam Med*. 2019;51(6):483-499. https://doi.org/10.22454/FamMed.2019.429968
8. Humbert AJ, Besinger B, Miech EJ. Assessing clinical reasoning skills in scenarios of uncertainty: convergent validity for a Script Concordance Test in an emergency medicine clerkship and residency. *Acad Emerg Med*. 2011;18(6):627-634. https://doi.org/10.1111/j.1553-2712.2011.01084.x
9. Bland AC, Kreiter CD, Gordon JA. The psychometric properties of five scoring methods applied to the Script Concordance Test. *Acad Med*. 2005;80(4):395-399. https://doi.org/10.1097/00001888-200504000-00019

Copyright © 2022 Bhardwaj et al. This is an open-access publication distributed under the terms of the Creative Commons Attribution license.

10. Kasten SJ, Kordorffer J, Downing S. Validity: giving meaning to assessments. In: Capella J, Kasten SJ, Steinemann S, Torbeck L, eds. *Guide for Researchers in Surgical Education.* Cine-Med; 2010:99-114.
11. Gagnon R, Charlin B, Lambert C, Carrière B, Van der Vleuten C. Script concordance testing: more cases or more questions? *Adv Health Sci Educ Theory Pract*. 2009;14(3):367-375. https://doi.org/10.1007/s10459-008-9120-8
12. Charlin B, Gagnon R, Lubarsky S, et al. Assessment in the context of uncertainty using the Script Concordance Test: more meaning for scores. *Teach Learn Med*. 2010;22(3):180-186. https://doi.org/10.1080/10401334.2010.488197
13. Bienstock JL, Katz NT, Cox SM, Hueppchen N, Erickson S, Puscheck EE. To the point: medical education reviews—providing feedback. *Am J Obstet Gynecol*. 2007;196(6):508-513. https://doi.org/10.1016/j.ajog.2006.08.021
14. Liaison Committee on Medical Education. *Data Collection Instrument for Full Accreditation Surveys in the 2021-22 Academic Year*. Association of American Medical Colleges/American Medical Association; 2021. https://lcme.org/wp-content/uploads/2022/07/2021-22_DCI_Full__2021-04-19.docx
15. Markert RJ, Barnes HV, Dunn MM, Goldenberg K, Hennessey JV. Comparing clerkship sites in a community-based medical school by evaluating students' undergraduate and postgraduate performances. *Acad Med*. 1993;68(4):298-300. https://doi.org/10.1097/00001888-199304000-00018
16. Mortaz Hejri S, Jalili M, Masoomi R, Shirazi M, Nedjat S, Norcini J. The utility of mini-Clinical Evaluation Exercise in undergraduate and postgraduate medical education: a BEME review: BEME Guide no. 59. *Med Teach*. 2020;42(2):125-142. https://doi.org/10.1080/0142159X.2019.1652732

Received: November 18, 2021
Accepted: July 7, 2022
Published: September 20, 2022

Copyright © 2022 Bhardwaj et al. This is an open-access publication distributed under the terms of the Creative Commons Attribution license.
